# Prickle is phosphorylated by Nemo and targeted for degradation to maintain Prickle/Spiny-legs isoform balance during planar cell polarity establishment

**DOI:** 10.1371/journal.pgen.1007391

**Published:** 2018-05-14

**Authors:** Giovanna M. Collu, Andreas Jenny, Konstantin Gaengel, Ivana Mirkovic, Mei-ling Chin, Ursula Weber, Michael J. Smith, Marek Mlodzik

**Affiliations:** Department of Cell, Developmental & Regenerative Biology and Graduate School of Biomedical Sciences, Icahn School of Medicine at Mount Sinai, New York, New York, United States of America; University of California San Diego, UNITED STATES

## Abstract

Planar cell polarity (PCP) instructs tissue patterning in a wide range of organisms from fruit flies to humans. PCP signaling coordinates cell behavior across tissues and is integrated by cells to couple cell fate identity with position in a developing tissue. In the fly eye, PCP signaling is required for the specification of R3 and R4 photoreceptors based upon their positioning relative to the dorso-ventral axis. The ‘core’ PCP pathway involves the asymmetric localization of two distinct membrane-bound complexes, one containing Frizzled (Fz, required in R3) and the other Van Gogh (Vang, required in R4). Inhibitory interactions between the cytosolic components of each complex reinforce asymmetric localization. Prickle (Pk) and Spiny-legs (Pk-Sple) are two antagonistic isoforms of the *prickle* (*pk*) gene and are cytoplasmic components of the Vang complex. The balance between their levels is critical for tissue patterning, with Pk-Sple being the major functional isoform in the eye. Here we uncover a post-translational role for Nemo kinase in limiting the amount of the minor isoform Pk. We identified Pk as a Nemo substrate in a genome-wide *in vitro* band-shift screen. *In vivo*, *nemo* genetically interacts with *pk*^*pk*^ but not *pk*^*sple*^ and enhances PCP defects in the eye and leg. Nemo phosphorylation limits Pk levels and is required specifically in the R4 photoreceptor like the major isoform, Pk-Sple. Genetic interaction and biochemical data suggest that Nemo phosphorylation of Pk leads to its proteasomal degradation via the Cullin1/SkpA/Slmb complex. dTAK and Homeodomain interacting protein kinase (Hipk) may also act together with Nemo to target Pk for degradation, consistent with similar observations in mammalian studies. Our results therefore demonstrate a mechanism to maintain low levels of the minor Pk isoform, allowing PCP complexes to form correctly and specify cell fate.

## Author summary

For functional tissues to form, individual cells must correctly orient themselves and function appropriately for their particular location in the body. The Planar Cell Polarity (PCP) complexes transmit one set of spatial cues by acting as signposts to mark direction across an epithelial layer. PCP signals can direct and coordinate cell differentiation, the behavior of groups of cells, or the orientation of individual cellular protrusions, depending on the tissue. PCP signals act as a polarization relay with two different complexes being positioned on opposite sides of each cell. This pattern of polarity is transmitted to neighboring cells and so extends across the tissue. In the fly eye, PCP signals control the differentiation of a pair of photoreceptors, R3 and R4, where the cell that is positioned closer to the dorso-ventral midline becomes R3. An excess of the PCP protein Prickle prevents the proper assembly of PCP complexes in the eye and so alters R3/R4 fate. Here we show that Nemo kinase is required in the R4 cell to phosphorylate Prickle and promote its degradation by the proteasome. Maintenance of low Prickle levels allows proper formation of PCP complexes, cell fate specification, and eye development.

## Introduction

Planar cell polarity (PCP) instructs tissue patterning in a wide range of organisms from *Drosophila* to humans, through input into cellular orientation across tissues, individual cell fate decisions, and the coordinated movement of groups of cells [1–8]. In the *Drosophila* eye, Frizzled core PCP signaling coordinates the cell fate decisions of individual photoreceptors, and their subsequent collective movements during ommatidial rotation, via asymmetric localization of two distinct membrane-bound complexes on opposite sides of a cell [1–7, 9]. The two core Fz/PCP pathway complexes comprise of Frizzled/Dishevelled/Diego (Fz/Dsh/Dgo) in one complex, and Van Gogh/Prickle (Vang/Pk) (Vang, also known as Strabismus/Stbm) in the other. These complexes are localized to opposite sides of the cell and stabilized intercellularly via the atypical cadherin Flamingo (Fmi) associating with both complexes [1–7]. Each component is highly conserved between *Drosophila* and vertebrates, and mutation of PCP genes in humans is linked to a range of diseases from *spina bifida* to polycystic kidney disease and epilepsy [10]. Feedback between the two complexes is essential to reinforce Wnt-induced cellular orientation bias [11–13] into coordinated tissue-wide polarity. Positive *inter*cellular interactions between transmembrane factors, Fz, Vang and Fmi, relay positional information and negative *intra*cellular interactions between cytosolic factors Pk and Dsh/Dgo enhance asymmetry on a cellular level [1, 3, 5, 7, 14]. In mammals there are four *prickle* genes and although there is only one *prickle* gene in *Drosophila* the range of Prickle functions are performed by distinct isoforms [15], Prickle (Pk), Spiny-legs (Pk-Sple) and PrickleM. Pk and Pk-Sple are the two functionally relevant isoforms during establishment of PCP. The balance between the two isoforms is tissue specific: Pk-Sple is the ‘major’ isoform in eyes and legs, and Pk the ‘major’ isoform in wings [15–17]. The precise balance has functional significance since the two isoforms can antagonize each other and/or the other’s function, although the underlying mechanism is not well understood [15–17]. Recent work has shown that this isoform balance is regulated transcriptionally at the tissue level in the wing, where Pk mRNA is present at 10-15-fold higher levels than Pk-Sple mRNA [17]. However, it is unclear how the balance is maintained in the eye, because it cannot be explained by transcriptional regulation: Pk mRNA is actually expressed at slightly higher levels than Pk-Sple mRNA [17], even though Pk-Sple is the major functional isoform here. When the Pk/Pk-Sple balance is perturbed it causes PCP defects, for example by overexpression of one isoform or isoform-specific alleles such as *pk*^*pk1*^, in which expression of only the Pk isoform is lost and Pk-Sple expression is unchanged [15–17]. Recent reports demonstrate that imbalance between the specific Pk and Pk-Sple isoforms causes seizures in *Drosophila* and, moreover, they suggest that disrupting *PRICKLE* genes underlies cases of epilepsy in humans [18, 19].

The *Drosophila* eye is a compound eye with ~800 individual ommatidia, each containing eight photoreceptor neurons (R1-8) arranged as a trapezoid [20–22](also Suppl S2 Fig). Chirality of the trapezoid is determined by positioning of the R3/R4 pair, whose fate is specified by Fz/PCP signaling. Fz activity is higher at the dorso-ventral midline (equator) of developing eye discs [20]. Consequently, for each R3/R4 pair the cell closer to the midline exhibits increased Fz activity, adopts the R3 fate, and signals to its neighbor to induce it as R4. Ommatidial preclusters then undergo a 90° rotation that is coordinated across the field by PCP activity, resulting in a line of symmetry around the equator. Disruption of PCP signaling causes chirality defects, whereby the R3/R4 fate decision becomes uncoupled from positional information or fails to be resolved [20]. PCP defects also involve misregulation of ommatidial rotation (OR) such that OR is no longer coordinated across the tissue [20–22].

Here, we report a new function for Nemo (Nmo) kinase, a classic ‘OR’ gene [23, 24], and demonstrate its role in regulating levels of the Pk isoform of *pk* via direct phosphorylation of Pk and its targeting for proteasomal degradation. Nmo is required in specific cells, the R4 cells, where the Pk isoform needs to be suppressed in the eye, and also in PCP mediated leg patterning, where Pk is also the minor functional isoform. Our results establish a new regulatory mechanism of PCP factors with tissue- and cell-specific regulation of core PCP protein degradation being coupled to PCP-mediated cell fate induction and function.

## Results and discussion

### Identification of Prickle as a Nmo kinase substrate

**Fig 1:**
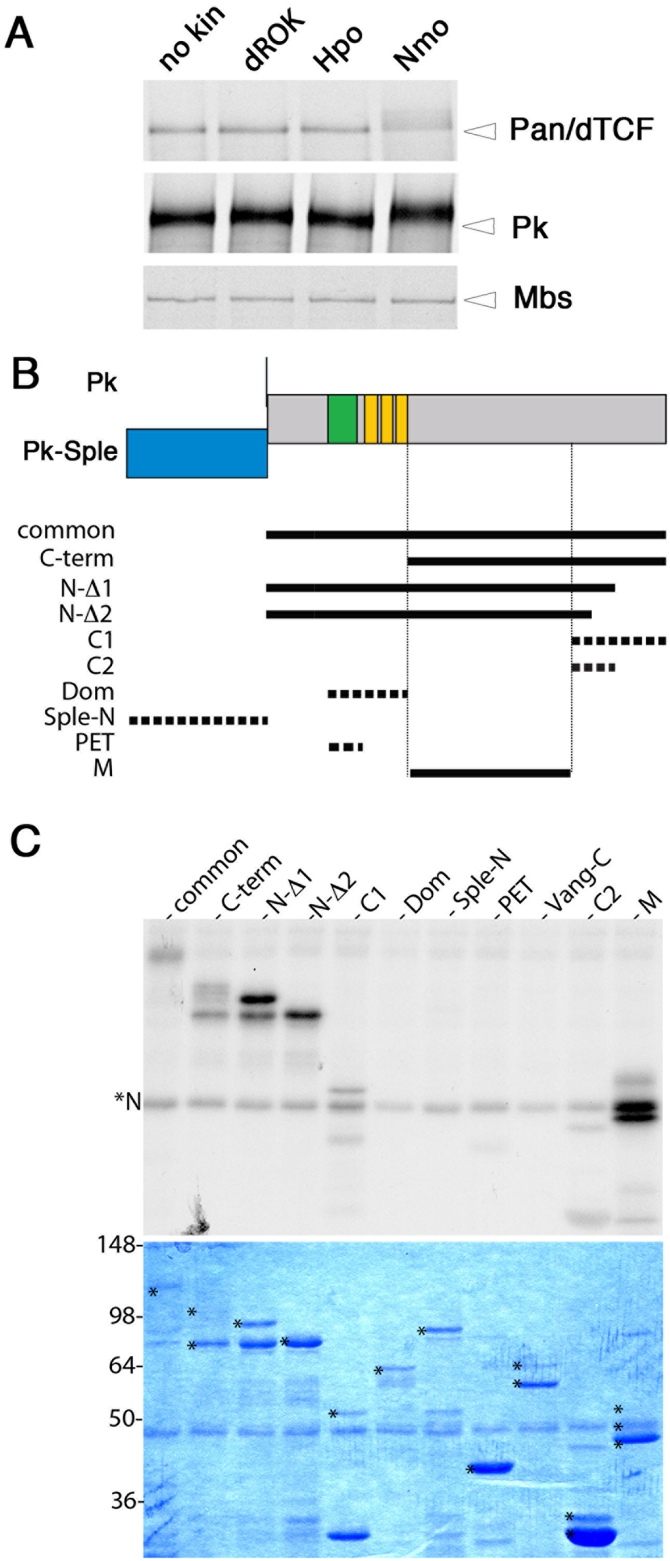
Nmo phosphorylates Pk. (**A**) Prickle is phosphorylated by Nmo kinase but not dROK or Hpo kinases. Band shift assay of *in vitro* translated Pk (common region), Pan/dTCF and Myosin binding subunit (Mbs). The open arrowhead denotes the size of the unphosphorylated form (compared to no kinase lane). Note the band shift of Pk. Pan and Mbs serve as positive and negative controls, respectively. (**B**) Schematic of Pk constructs used in this study. The Pk protein scheme is shown above with the Pk-Sple N terminus (blue), short Pk N terminus as black line, PET domain (green) and three LIM domains (yellow). Constructs as denoted here are indicated by thick black lines: common (sequence shared between Pk and Pk-Sple isoforms); C-terminus; N-Δ1; N-Δ2 (both C-terminal deletions as indicated); C1; C2; Dom (PET and LIM domains); Sple N-terminus (Pk-Sple specific N-terminal extension); PET domain; M (Middle sequence) (Methods for sequence details). Full lines indicate fragments that are phosphorylated by Nmo, dashed lines indicate unphosphorylated fragments. (**C**) *In vitro* kinase assay gel using purified Nmo kinase and *in vitro* translated Pk fragments (from panel **B**). Upper panel; radiograph showing phosphorylation; autophosphorytion of Nmo is denoted by *N. Vang C-term is used as negative control (also [24]). Common, C-term, N-Δ1, N-Δ2, and M fragments are phosphorylated by Nmo. Corresponding Coomassie-stained gel is shown below—full-length fragments of individual constructs are indicated by *.

In order to better understand how Nmo acts as a PCP effector in the eye, we performed a genome-wide, gel-shift based screen for novel Nmo kinase substrates, similarly to our recent studies identifying novel dROK and Hpo substrates [25, 26]. In brief, pooled cDNA clones were *in vitro* translated and labeled with ^[35]^S-Methionine, and then incubated with purified Nmo in the presence of unlabeled ATP. Reduced mobility on Anderson gels was used as the criterion to select candidate substrates. Surprisingly, one of the candidates identified in this screen was Prickle, an ‘upstream’ core component of PCP complexes. Nmo, but not dRok or Hpo kinases, was able to induce a band shift of Prickle (Fig 1A), as well as the positive control Pan/dTCF [27], but not the negative control, Mbs (Fig 1A and Suppl. S1 Fig). Band shift assays of cell culture extracts confirmed that Nmo kinase activity reduces the mobility of Prickle (Suppl. S1 Fig).

Using purified Nmo kinase we performed *in vitro* kinase assays and determined that Nmo directly phosphorylated the common region of Pk. The two main Prickle isoforms required for PCP, Pk and Pk-Sple, are largely identical except for an extended Pk-Sple N-terminal region (outlined in Fig 1B). To identify the Nmo phosphorylation site(s) within the common Pk sequence, we designed a series of deletion constructs (outlined in Fig 1B) with the Vang C-term included as a negative control [24]. Through comparison of the phosphorylation of these constructs in *in vitro* kinase assays, the phosphorylation sites were mapped to a middle region of the common Pk sequences (fragment M, Fig 1B and 1C). Nmo did not phosphorylate the Sple N-terminus, PET and LIM domains, or the region within the Pk C-terminus required for binding to Vang (contained within fragment C1 [28]).

Our previous studies had suggested a model whereby Vang recruited Nmo to the membrane in ‘mature’ ommatidial clusters, where it acted as an effector and phosphorylated β-catenin to promote cluster rotation [24]. Prickle being a Nmo substrate raised the possibility that Nmo had an earlier role in regulating the PCP complexes themselves. Although the zebrafish *nmo* homolog, *nemo-like kinase* had been known to genetically interact with the Wnt/PCP pathway during the coordinated movements of convergent extension in the embryo [29], our result is the first indication that *nmo* directly affects the core PCP factors during the establishment of PCP itself.

### *nmo* genetically interacts with the *pk* but not *sple* isoform-specific mutant

**Fig 2:**
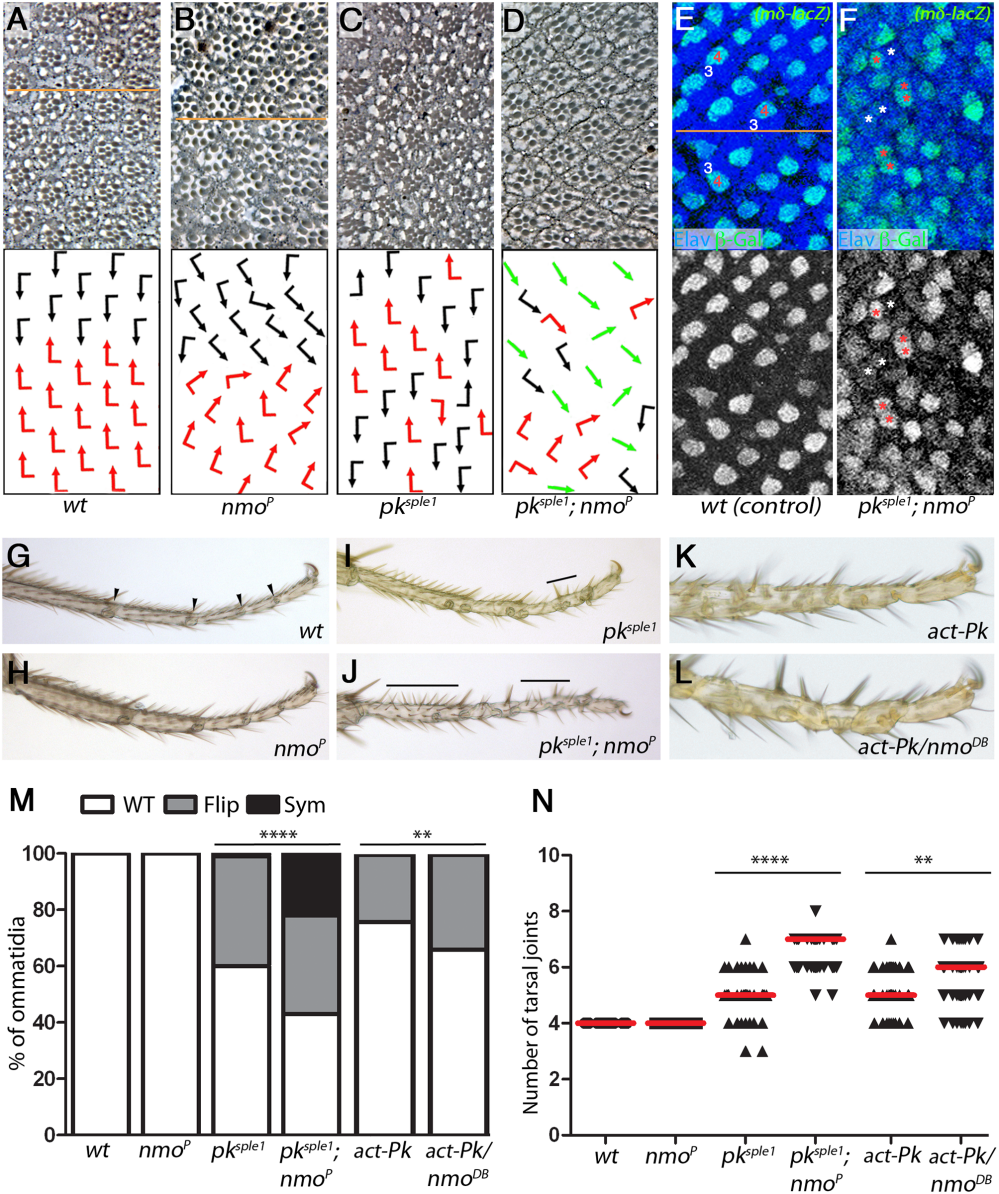
*nmo* enhances the PCP defects of *pk*^*sple1*^ mutants. (**A—D**) *nmo* genetically interacts with *pk*^*sple1*^ in the eye. Tangential sections of adult eyes of the indicated genotype are shown with corresponding schematics below. Chiral ommatidia are depicted as black (dorsal) or red (ventral) flagged arrows and symmetrical clusters are green straight arrows (also Suppl. S2 Fig). The dorso-ventral midline (line of mirror symmetry) can be seen in *wt* (**A**) and *nmo*^*P*^ (**B**) mutants, whereas chiral forms are intermixed in homozygous *pk*^*sple1*^ mutants (**C**). In *pk*^*sple1*^; *nmo*^*P*^ double mutants (**D**), the proportion of chirality defects significantly increases with many symmetrical clusters (quantified in **M**). (**E-F**) Detection of symmetrical clusters in larval eye discs. Immunofluorescence of an R4-specific molecular marker (*mδ-LacZ*, green) and a pan-neuronal marker (Elav, blue). Examples of *mδ-LacZ*-negative cells are marked in white (R3 or *) and *mδ-LacZ*-positive cells are marked in red (R4 or *). (**E**) Wild-type: Note regular arrangement of β-gal-positive cells with one cell located at the polar side/R4 of each cluster (equator is in center of panel, see Suppl. S2 Fig for detail of frame positioning). (**F**) R3/R4 fate is affected in *pk*^*sple1*^; *nmo*^*P*^ animals. Note disorganization of β-gal-positive cell pattern, and the presence of clusters with either two or none *mδ-LacZ* positive cells (symmetrical clusters). (**G-J,N**) *nmo*^*P*^ enhances the *pk*^*sple1*^ PCP defects in legs. Tarsal segments of adult legs of the indicated genotype are shown. *wt* and *nmo*^*P*^ mutant legs have four tarsal joints (**G,H,N**) joints are marked with arrowheads in **G**). Extra joints form in *pk*^*sple1*^ mutants, and incomplete joint tissue can be seen within tarsal segments (**I,N**). The line shows a region of reversed polarity (bristles point proximally). The median joint number increases in *pk*^*sple1*^; *nmo*^*P*^ double mutants (**J**) from five to seven (**N**), and the extent of polarity reversal is increased (lines in **J**). (**K-L**) Loss of *nmo* function enhances the *act-EGFP-Pk* gain of function leg phenotype. Extra joint tissue forms in *act-EGFP-Pk* tarsal segments (**K**), and joint number further increases in *act-EGFP-Pk/nmo*^*DB*^ animals (**L**; quantified in panel **N**: median joint number increases from five to six). (**M**) Quantification of chirality defects (**** *P*< 5E^-24^, ** *P*<0.005, Chi-squared test n>200). (**N**) Quantification of tarsal joints (**** *P* = 5E^-24^, ** *P*<0.0001, Mann Whitney U test n>54).

Given that Nmo phosphorylated a region that is shared between the two *prickle* isoforms, Pk and Pk-Sple, we wanted to determine whether one or both isoforms were functionally affected *in vivo*. We performed genetic interaction assays with *nmo* mutants and isoform specific alleles, *pk*^*pk1*^ and *pk*^*sple1*^ (loss of function, LOF) or over-expressed individual Pk and Pk-Sple isoforms (gain of function, GOF). We first examined the interaction between *nmo* and *pk*^*sple1*^ in the eye. Wild-type and *nmo*^*P*^ hypomorphs show wild-type ommatidial chirality, in addition to the previously-described underrotation in *nmo* mutant clusters (Fig 2A, 2B and 2M) [23, 24, 30]. *pk*^*sple1*^ clusters adopt almost random chirality, whereby the R3/R4 fate decision is resolved, but the dorsal vs ventral chiral arrangements are intermixed, termed ‘chirality flips’, and R3/R4 fate is uncoupled from dorso-ventral positioning (Fig 2C and 2M). However, in the *pk*^*sple1*^; *nmo*^*P*^ double mutant, a significant proportion of achiral, symmetrical clusters were observed; a phenotype rarely observed in either single mutant (Fig 2D and 2M, note over 20-fold increase in symmetrical clusters—see Suppl. S2A Fig for schematic of photoreceptor arrangement). We confirmed the achiral nature of the clusters by immunostaining larval eye discs using the *mδ-LacZ* construct, which in wild-type specifically labels the R4 precursor [31, 32] (Fig 2E and 2F and Suppl. S2B Fig for overview). Clusters with two negative or two positive cells can often be seen in the double mutant, but not wild-type, where there is a regularly-spaced array of one β-gal positive cell per cluster (Fig 2E and 2F). Such symmetrical clusters are considered the strongest PCP defect as they completely fail to resolve the R3/R4 fates, indicating an inability to establish PCP-mediated cell fate differences at all [20, 22, 33]. Interestingly, symmetrical clusters were also observed in *nmo*, *fz* double mutant clones [34], but this was neither commented upon nor followed up on in that study.

To further define the genetic interaction between *pk*^*sple1*^ and *nmo*^*P*^, we analyzed the PCP phenotype in the tarsal region of the leg. *pk*^*sple1*^ mutants display supernumerary tarsal joints with altered bristle polarity (Fig 2G, 2I and 2N) [15]. This results in spiny-looking legs, giving the allele its name. Compared to *pk*^*sple1*^ single mutants, joint number significantly increased in *pk*^*sple1*^; *nmo*^*P*^ double mutants (Fig 2I, 2J and 2N). As the only isoform expressed in *pk*^*sple1*^ adults is Pk, we confirmed the genetic interaction in a Pk GOF assay. Consistent with the notion that eye patterning is very sensitive to Pk isoform levels, even the low level overexpression of Pk (via direct *act-Pk*, without Gal4-associated amplification) is sufficient to unsettle the balance between Pk and Pk-Sple and cause defects: *act-EGFP-Pk* animals [35] displayed predominantly ‘flips’ in the eye (Fig 2M and Suppl. S2G Fig). The chirality defects in *act-EGFP-Pk* animals were dominantly enhanced by *nmo*^*DB/+*^ (Fig 2M and Suppl. S2G and S2H Fig). As *act-EGFP-Pk* is expressed at low levels throughout the animal, we also analyzed the leg phenotype. Consistent with the eye results, loss of *nmo* function enhanced the ectopic joint phenotype associated with *act-EGFP-Pk* (Fig 2K, 2L and 2N; note that like the eye the PCP leg patterning is also sensitive to the Pk/Pk-Sple balance). In contrast, we did not observe PCP defects in wings of *pk*^*sple1*^; *nmo*^*P*^ mutants or *act-EGFP-Pk* and *act-EGFP-Pk/nmoDB* animals; all wings displayed a wild-type appearance (Suppl. S3 Fig, note Pk is the major isoform in the wing).

Examining whether there was an interaction between *pk*^*pk1*^ (where Pk-Sple is the isoform expressed) and *nmo* in the eye, we detected no chirality defects (Suppl. S2C, S2D and S2I Fig, note that *pk*^*pk1*^; *nmo* double mutant ommatidia displayed only the expected *nmo* rotation phenotypes). Moreover, in GOF scenarios with overexpressed Pk-Sple, no interaction between *nmo* and Pk-Sple was observed in either tissue studied (Suppl. S4 Fig), although *act-EGFP-Sple* wings did have a strong PCP phenotype [17, 35] (Suppl. S4C–S4E Fig). Importantly, in a *pk* null background (*pk*^*pk-sple13*^, with neither Pk or Pk-Sple isoforms expressed), we did not detect an increase in symmetrical cluster formation in *pk*^-^; *nmo* double mutant ommatidia (Suppl. S2E, S2F and S2I Fig). The double mutant phenotype resembles that of *pk* null eyes, further reinforcing the notion that Nmo acts specifically on the Pk isoform, and thus if both isoforms are absent (as with the null allele) Nmo has no PCP substrate upon which to act. Collectively, the above data suggest that Nmo phosphorylates the Pk isoform of the *pk* gene, which has functional consequences in eyes and legs; tissues where both isoforms are expressed and Pk-Sple is the major protein isoform. Given the importance of the Pk/Pk-Sple balance, Nmo might be required to repress the Pk isoform in these tissues.

### Phosphorylation by Nmo limits Prickle activity

**Fig 3:**
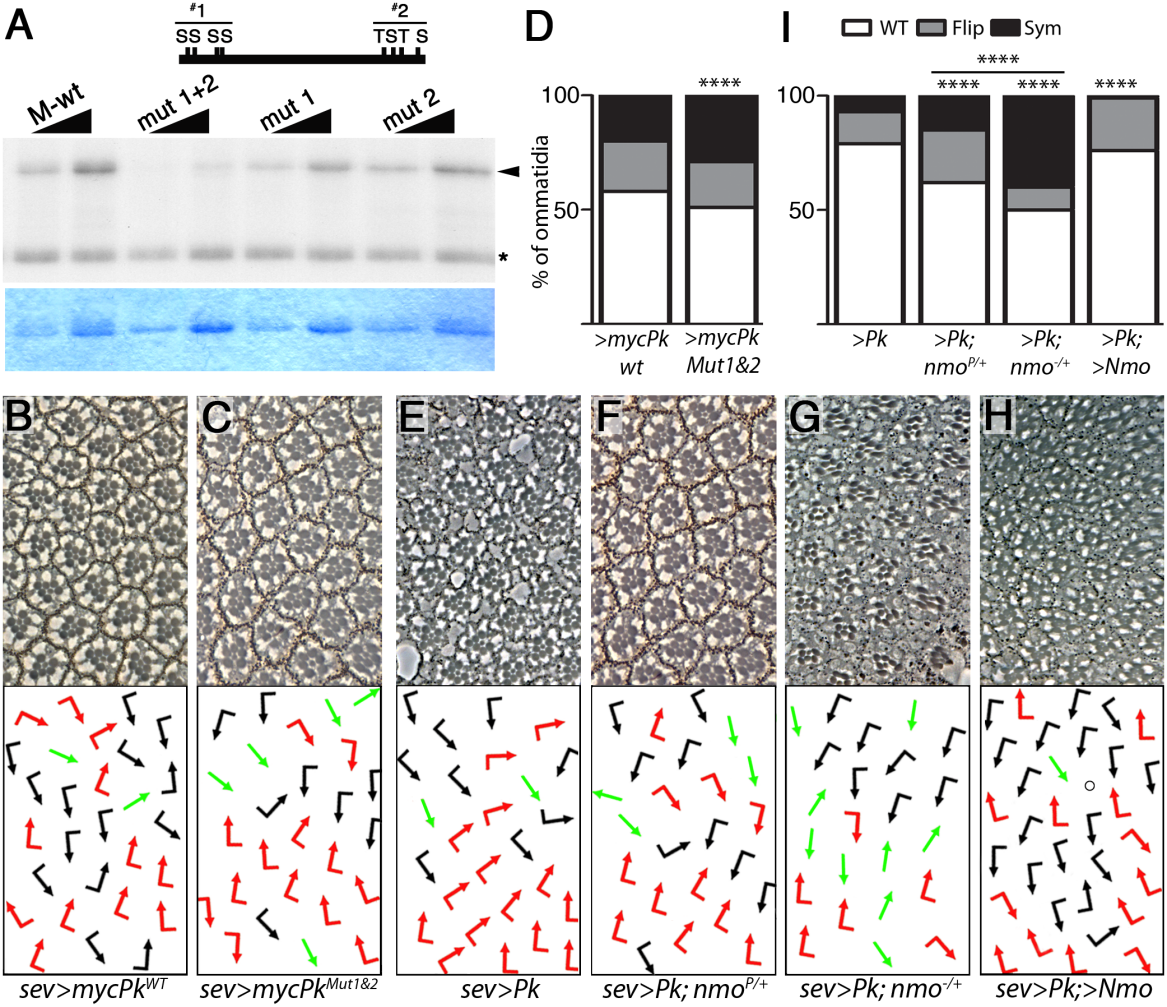
Nmo limits Pk^Pk^ activity. (**A**) Identification of Nmo phosphorylation sites. *In vitro* kinase assay with increasing amounts of M fragment (see also Fig 1) in which the first or second cluster of MAPK consensus sites, (potential Nmo target sites), or both, are mutated. Positions of the Nmo S/T phosphorylation sites are indicated in above schematic of fragment with S and T, respectively (the S/T residues in Pk are for cluster 1: S515, S519, S595, and S599, and for cluster 2: T708, S725, T737, and S762). Upper panel; radiograph showing phosphorylated Pk fragments (arrowhead; * denotes Nmo autophosphorylation), lower panel; Coomassie-stained gel. Note that phosphorylation is significantly reduced only when both clusters are mutated. (**B-D**) Pk lacking both clusters of Nmo phosphorylation sites shows a stronger phenotype. *sevenless(sev)*-*Gal4* driven Pk overexpression (myc-Pk-WT; panel **B**) displays a gain of function phenotype with both flips and symmetrical clusters. The phenotype is more severe when a Pk construct in which both Nmo consensus site clusters have been mutated (myc-Pk-Mut; note increased symmetrical clusters, panel **C**) is expressed. Both transgenes are inserted in the same attP site and thus expressed at same levels; quantified in panel **D** (**** *P*<0.00005 Chi-squared test, n>300). (**E-I**) Dose-dependent effect of Nmo on the *sev*-*Gal4* driven Pk gain-of-function phenotype. *sev*-driven Pk expression causes chirality defects (**E**). (**F-G**) Reduction of Nmo function through either one copy of the hypomorphic allele, *nmo*^*P/+*^ (**F**), or the null allele, *nmo*^*DB/+*^ (**G**) enhances these PCP defects, particularly number of symmetrical clusters (quantified in panel **I**); in contrast Nmo co-overexpression with Pk, (*sev>Pk*, *>Nmo*; panel **H**) suppresses the Pk-induced formation of symmetrical clusters (quantified in panel **I**; *P*<0.0002, circle represents cluster with R-cell loss). (**I**) Quantification of chirality defects (****P<0.0005, Chi-squared test n>300).

The genetic interaction between *pk*^*sple1*^ and *nmo*^*P*^ being similar to that between Pk over-expression and *nmo* LOF (Fig 2M and 2N) suggests that the phenotype of the *pk*^*sple1*^; *nmo*^*P*^ double mutants results from increased Pk activity and/or amount. We therefore proceeded to examine the effect of Nmo phosphorylation on Pk activity. The sequence of the Nmo target fragment of Pk, region M (Fig 1B and 1C), contains two clusters of 4 potential MAPK phosphorylation sites (Fig 3A; Nmo is a member of the MAPK family). All 4 serine/threonine residues in each cluster were mutated to alanine to create phospho-mutant cluster 1, cluster 2, or a construct with all 8 sites mutated (mut1+2; Fig 3A). These fragments were tested in *in vitro* kinase assays, which revealed that mutations of either cluster alone had little effect; but when both clusters were mutated phosphorylation was markedly reduced (Fig 3A). These data identify two clusters of MAPK consensus sites within the common M fragment of Pk as direct phosphorylation targets of Nmo.

To investigate the effect of these Nmo phosphorylation sites *in vivo*, we generated transgenic flies of either wild-type myc-Pk, or “phospho-mutant” myc-Pk (Pk^WT^ and Pk^Mut1&2^, where both clusters of Nmo phosphorylation sites were mutated to alanine) (Fig 3A and Methods). Pk overexpression in both cells of the R3/R4 precursor pair during PCP signaling (under *sevenless*-Gal4 control: *sev>Pk*; [28, 36]) produced a phenotype with rotation and chirality defects (Fig 3B and 3D; chirality defects were mainly ‘flips’, although some symmetrical clusters were observed). Comparing Gal4-driven expression of wild-type Pk and phospho-mutant Pk (Pk^mut1+2^) the phospho-mutant displayed more severe phenotypes with an increase in chirality defects and particularly symmetrical clusters (Fig 3B and 3D; both transgenic constructs were inserted in the same genomic *att*-site and are thus transcriptionally expressed at equal levels; Methods).

The above data mimic the effect of *nmo* loss-of-function on wt-Pk (Fig 2M) and below, corroborating the notion that the phosphorylation event causes a post-transcriptional reduction in Pk activity/levels. We modulated levels of Nmo and assessed the effects in the *sev*-driven Pk GOF assay in the eye. We used either *nmo* loss-of-function alleles (*nmo*^*P*^ and *nmo*^*DB*^ alleles: Fig 3F and 3G, hypomorphic and null, respectively) or Nmo co-overexpression (Fig 3H) [24]. There was a dose-dependent effect of loss of *nmo* function on the *sev*>*Pk* phenotype. Chirality defects increased in *nmo* heterozygotes with an increased number of symmetrical clusters in particular (Fig 3E–3G and 3I). Conversely, Nmo co-overexpression with Pk suppressed the *sev*>*Pk* phenotype, markedly reducing the number of symmetrical clusters (Fig 3E, 3H and 3I). Consistently with this, increasing the levels of Pk in a *pk*^*sple1*^ background causes a synergistic increase in symmetrical clusters, similarly to Nmo LOF (Suppl. S5B and S5C Fig). These data are consistent with a hypothesis that Nmo function is required to limit Pk activity or levels. Consistent with our earlier results, we did not detect an effect of *nmo* LOF on the Pk-Sple isoform overexpression phenotype (Suppl. S4C–S4I Fig) or differences in the activity of wild-type Pk-Sple compared to to Pk-Sple^Mut1&2^ (*sev-Sple*^*WT*^ *vs sev-Sple*^*Mut1&2*^, the equivalent mutations to Pk^Mut1&2^; Suppl. S5E and S5F Fig). Moreover, mutation of the Nmo sites in Pk-Sple, does not affect the ability of the Pk-Sple isoform to rescue the chirality defects present in the *pk* null mutant (Suppl. S5G Fig). We did not observe a difference in the activity levels of wild-type and phospho-mutant Pk in the wing either (nub>Pk^WT^ and >Pk^Mut1&2^; Suppl. S5D Fig).

Collectively, these results support a model in which Nmo acts primarily on Pk to maintain a tissue-specific balance of Pk/Pk-Sple activity in the eye and leg, where both are expressed with Pk-Sple being the major isoform. Therefore, post-translational regulation of Pk acts in the eye, in addition to the transcriptional control of isoform expression previously described for the wing [17]. Although the Nmo phosphorylation sites are shared between all *pk* isoforms, we have no evidence to suggest that the Pk-Sple isoform is affected. The Pk and Pk-Sple isoforms are known to form different protein complexes [37], potentially explaining this disparity. It remains possible that Nmo does also phosphorylate Pk-Sple, but that Pk-Sple phenotypes are not affected by Nmo, potentially because the Pk-Sple specific N-terminus interferes with phosphorylation and/or masks the biological read-out.

### *nmo* is required specifically in the R4 photoreceptor

**Fig 4:**
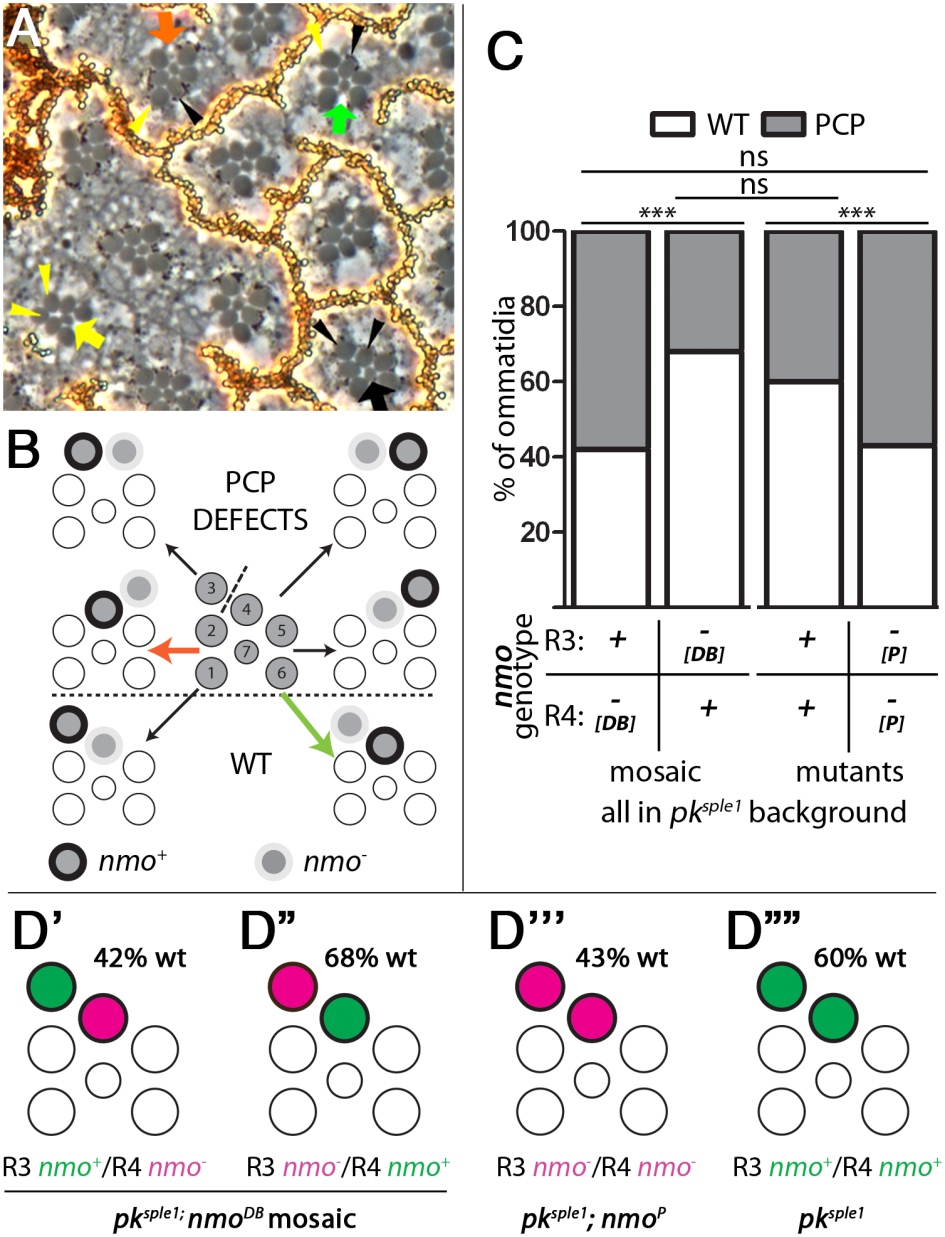
*nmo* is required in the R4 photoreceptor for correct chirality establishment. Clonal analysis of *nmo* function in a *pk*^*sple1*^ background, all cells are mutant for *pk*^*sple1*^, and marked clones are also mutant for the *nmo* null allele, *nmo*^*DB*^ (marked by lack of pigment granules, small black dots adjacent to rhabdomeres—examples of *nmo*+ Rs are labelled with black arrowheads and *nmo*- Rs are labelled with yellow arrowheads in A). Only the genotype of the R3-R4 pair was scored in pairs in which R3 and R4 were of different genotypes. (**A**) Representative image of a tangential section of an adult mosaic eye; black arrow points to a correctly-oriented cluster in which R3 and R4 exhibit pigment granules and are thus *nmo*+ (or *wt* for *nmo*, marked by black arrowheads); yellow arrow points to a symmetrical cluster in with both R3 and R4 lack pigment granules and therefore are *nmo-* (marked by yellow arrowheads); green arrow points to a correctly-oriented cluster in which *nmo* function is only present in R4 (R3: *nmo*^-^/R4: *nmo*^+^); red arrow points to an incorrectly-oriented cluster in which *nmo* function is only present in R3 (R3: *nmo*^+^/R4: *nmo*^-^). (**B**) Schematic of mosaic analysis. Clusters in which *nmo* function is present in R3 only (pigment is represented by black circle, left column), or R4 only (right column), can develop in a wild-type orientation (below) or can exhibit PCP defects (above line). Red arrow points to an incorrectly-oriented cluster with *nmo* function in R3 only (as in real cluster in panel **A**) and green arrow points to a correctly-oriented cluster with *nmo* function in R4 (as in panel **A**). (**C**) Quantification of wt and PCP defects in clusters in which only R3 has *nmo* function (first column) compared to those with *nmo* function in R4 (second column). There is a significant increase in the proportion of WT clusters when *nmo* is present only in R4, compared to only in R3 (*P* = 0.0022, Fisher’s exact test n = 140 pairs) For comparison, the data from Fig 2M are also shown; *pk*^*sple1*^ (third column) and *pk*^*sple1*^; *nmo*^*P*^ double mutants (fourth column). There is no significant difference between *pk*^*sple1*^ mutants and loss of *nmo* function in R3 only, or between *pk*^*sple1*^; *nmo*^*P*^ double mutants and loss of *nmo* function in R4 only (*P*>0.05). (**D**) (**D′**) In ommatidia that have Nmo function in R3 only (R3 *nmo*^+^/R4 *nmo*^-^) in *pk*^*sple1*^ background, 42% ommatidia develop as wild-type clusters with correct chirality. (**D′′**) In ommatidia that have Nmo function in R4 (R3 *nmo*^-^/R4 *nmo*^+^) in *pk*^*sple1*^ background, 68% develop as wild-type clusters with correct chirality, resembling the *pk*^*sple1*^ single mutant background. (**D′′′**) In *pk*^*sple1*^; *nmo*^*P*^ double mutants with both R3 and R4 lacking Nmo function (R3 *nmo*^-^/R4 *nmo*^-^) 43% of clusters develop with correct chirality, comparable to genotype in panel (**D′**). (**D′′′′**) In *pk*^*sple1*^ single mutants, 60% of clusters develop with correct chirality. Compare panels **D′** and **D′′′**: when R4 lacks Nmo function, the proportion of wild-type clusters is similar, irrespective of the genotype of R3. This suggests that Nmo acts in R4 during chirality establishment/R3 vs R4 specification. Compare panels **D′′** and **D′′′′**: when R4 has Nmo function, the proportion of wild-type clusters is similar to the *pk*^*sple1*^ single mutant, irrespective of the genotype of R3. Together this suggests that *nmo* is only required in R4 for correct chirality. Green denotes Nmo function and magenta denotes lack of Nmo function. Note, in all cases described, all cells are mutant for *pk*^*sple1*^.

Based on these results we would predict Nmo to be required in the polar R4 precursor to limit Pk activity, because the *pk* gene (the Pk-Sple isoform) is required in the polar cell to establish PCP complexes and direct proper cell fate [28]. Genetic mosaic analysis is highly useful to determine which cell of the R3/R4 precursor pair requires an individual core PCP gene [14, 28, 34, 38, 39]. As *nmo* mutants show frequent chirality defects only in a *pk*^*sple1*^ background rather than wild-type, we performed mosaic analysis in the *pk*^*sple1*^ genetic background. Specifically we induced clones of *nmo*^-^ cells in *pk*^*sple1*^ eyes and analyzed R3/R4 pairs that were bisected by the clonal boundary; one cell of the R3/R4 pair was *nmo*^+^ and the other *nmo*^-^ (Methods). Clones of *nmo*^-^ mutant cells were marked by absence of pigment (Fig 4A and 4B shows schematic and example image). If *nmo* were specifically required only in one cell of the pair, for instance the R4 precursor, then we would expect to see more clusters developing with wild-type chirality when *nmo* function is removed from the other cell, R3, than from R4. Strikingly, when *nmo* function was only removed from R4, ommatidia displaying wild-type chirality were markedly reduced (to 42%), very similar to the fully double mutant *pk*^*sple1*^; *nmo*^*P*^ eyes (Fig 4C and 4D’). In contrast, when only R3 was *nmo*^-^, the proportion of wild-type clusters was 68%, very similar to *pk*^*sple1*^ single mutant eyes (Fig 4C and 4D”). These data indicate that this *pk*-associated *nmo* function is specifically required in the R4 cell. This was confirmed when the chirality defect ratios were compared with the *pk*^*sple1*^ single and *pk*^*sple1*^; *nmo*^*P*^ double mutants (Fig 4C, 4D”’ and 4D‘”’). Thus the mosaic analyses suggested that for chirality establishment and core PCP function, it is the presence of Nmo in R4 that is required. The combined mosaic analyses of *nmo* (this work) and our previous study of *pk* [28] demonstrate that Nmo is required in the same cell as the *pk* gene to reduce the activity/levels of Pk in order for functional PCP complexes to be established. Compare this result to the genetic requirement of *nmo* in ommatidial rotation, when it functions as an effector of PCP, and is required in all R-cells and even cone cells [24, 40]. Together these results demonstrate a spatially- and temporally-distinct role for *nmo* in regulating Pk in core PCP complex establishment in R4, and then subsequently acting as an effector downstream of the core PCP complexes throughout the ommatidial cluster to regulate ommatidial rotation.

### Nmo promotes Pk degradation by the proteasome

**Fig 5:**
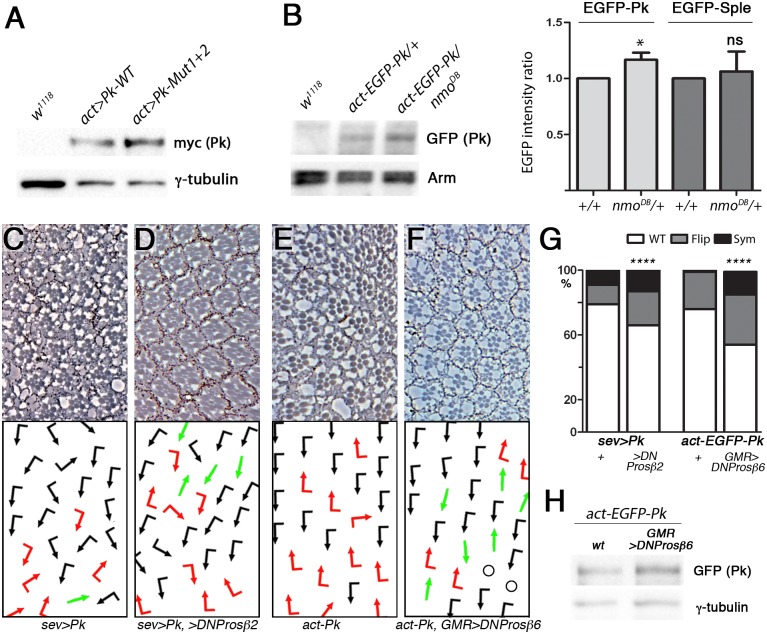
Nmo regulates Pk levels via proteasome-mediated degradation. (**A**) Loss of *nmo* phosphorylation sites increases the Pk protein level in eye discs. Lysates from myc-Pk-WT- or myc-Pk-Mut-expressing eye discs were immunoblotted using myc and γ-tubulin (control) antibodies. *w*^*1118*^ discs were used as a control. (**B**) Loss of *nmo* function increases Pk protein level in eye discs. The relative amount of EGFP-Pk protein in a *wt* or *nmo*^*DB/+*^ background was calculated and normalized to Arm levels. A representative blot is shown, data from four independent experiments are quantified in graph to the right. Reduction of *nmo* function increases the amount of EGFP-Pk protein, but not that of EGFP-Sple (see Suppl. S6 Fig for blot; paired t-test, * *P* = 0.038, ns *P*>0.05). (**C-F**) Eye phenotypes of *sevGal4*, *UAS-Pk* (*sev>Pk*) and *act-EGFP-Pk* co-expressing dominant negative (DN) proteasome components. The *sev*>*Pk* phenotype (**C**) is enhanced by co expression of DNProsβ2 (**D**). The *act*-*EGFP-Pk* phenotype (**E**) is enhanced by *GMR>DNProsβ6* co-expression (**F**). Note the increase in symmetrical clusters. (**G**) Quantification of eye phenotypes (****P<0.0001, Chi-squared test n>300). (**H**) Inhibition of proteasome function increases Pk protein level in eye discs. Lysates from eye discs expressing *act*-*EGFP-Pk* in either a *w*^*1118*^ or *GMR*> *DNProsβ6* background were immunoblotted using GFP and γ-tubulin (control) antibodies.

One possibility of how Nmo could limit Pk activity is to regulate the levels of the Pk isoform, thereby preventing excess Pk disrupting the Pk/Pk-Sple isoform balance. To examine *nmo* loss-of-function effects on Pk levels, we performed Western blots with larval eye disc lysates. We first compared the levels of wild-type Pk and Pk^Mut1&2^ that were expressed under *actin*-Gal4 control. Compared to wild-type myc-Pk, mutation of the Nmo phosphorylation sites resulted in an increased protein level, as quantified as signal ratio of myc:gamma tubulin antibodies (Fig 5A and Suppl. S6D and S6H Fig). We then compared levels of *act*-EGFP-Pk in either wild-type or *nmo*^DB/+^ backgrounds (Fig 5B and Suppl. S6B and S6I Fig). *nmo* loss-of-function resulted in an increase in EGFP-Pk levels (Fig 5B), similarly to mutation of Nmo phosphorylation sites (Fig 5A). As *nmo* is only expressed in a stripe posteriorly to the furrow [41], and its effect on Pk is specifically required during R3/R4 specification, there is a sizeable amount of *act*-EGFP-Pk that is unaffected, explaining the subtle increase, which is nevertheless significant. Conversely and as a specificity control, we did not detect a similar change in EGFP-Sple levels in a *nmo*^*DB/+*^ background, confirming that *nmo* acts specifically on the Pk isoform (Fig 5B and Suppl. S6A–S6C Fig). These data are consistent with Nmo functioning to maintain lower levels of the Pk isoform, thus limiting the Pk/Pk-Sple ratio. Given the increase in Pk^Mut1&2^, which is expressed from a transgene lacking endogenous 3’ UTR, under control of Gal4/UAS system, this also indicated that Nmo regulates Pk at the post-translational level.

Degradation by the proteasome could be a means to regulate Pk levels. We examined this hypothesis by first co-expressing a dominant negative (DN) form of the proteasome 20S β2 subunit, Prosbeta2 [42–44] along with Pk under *sev*-Gal4 control and analyzing the adult phenotype. DNProsbeta2 expression synergized with the Pk GOF phenotype to enhance chirality defects, particularly symmetrical clusters (Fig 5C, 5D and 5G). In a complementary approach, we used the milder *act*-EGFP-Pk phenotype and examined the effect of another DN proteasome component—this time β6 (Prosbeta6) [42–44], under the control of *GMR*-Gal4, which is expressed in all post-mitotic, differentiating cells in the eye. We again saw an increase in chirality defects, and of symmetrical clusters in particular (Fig 5E–5G). Moreover in this scenario, we also saw an increase in *act*-EGFP-Pk protein levels (Fig 5H and Suppl. S6E Fig). In both cases, the control animals, with mildly reduced proteasome function alone, did not induce chirality defects (Suppl. S6F and S6G Fig).

### The SCF complex promotes Pk degradation

**Fig 6:**
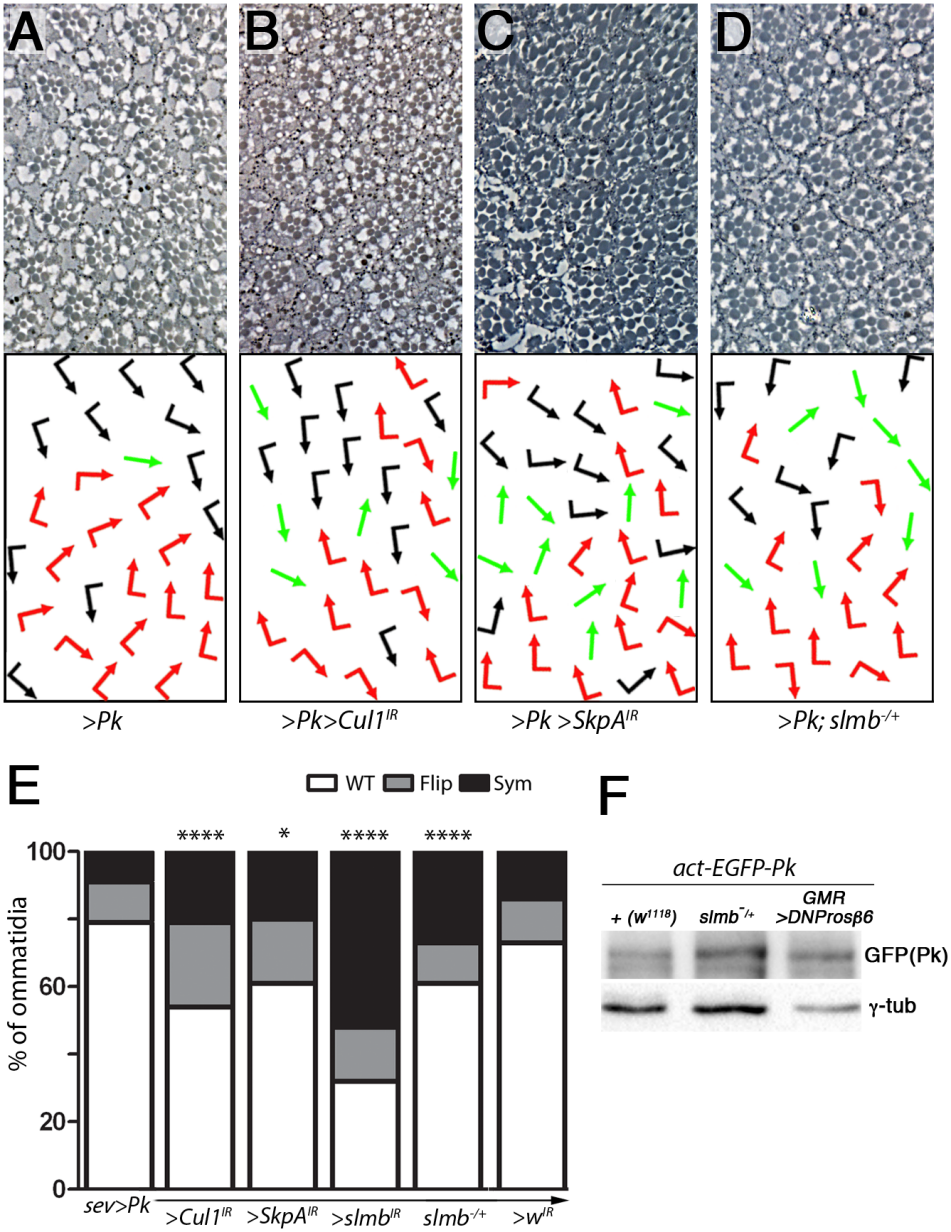
The Cul1/SkpA/Slmb SCF complex promotes Pk degradation. (**A-D**) Eye phenotypes of *sevGal4*, *UAS-Pk* (*sev>Pk*) in the indicated backgrounds of reducing levels of components of SCF-E3 ligase complex (Cul1, SkpA, and Slmb). Knockdown of *Cul1* (**B**) and *SkpA* (**C**) as well as *slmb*^*+/-*^ background (**D**) all enhance the Pk gain-of-function phenotype (**A**), quantified in (**E**) also quantified are *white* RNAi as a negative control and *slmb* knockdown (See Fig 7 and Suppl. S7 Fig for additional sections). In each genotype *n*>200 and *P*<0.02, 0.001, and 0.0005 in *, ***, and ****, respectively). (**F**) Reduction of Slmb function increases Pk protein level in eye discs. Lysates from eye discs expressing *act*-EGFP-Pk in either a *w*^*1118*^ or *slmb*^*+/-*^ background were immunoblotted using GFP and γ-tubulin (control) antibodies. *GMR*>*DNProsbeta6* is included for comparison.

It has been suggested that Pk levels are constitutively regulated by the Cullin1/ SkpA/Supernumary limbs (Slmb) SCF E3-ubiquitin ligase complex in *Drosophila* wings [45, 46]. Consequently, we reasoned that the SCF complex might be operating in the eye to regulate Pk levels through ubiquitination and promoting subsequent proteasomal degradation of the Pk isoform. To test this hypothesis, we reduced the activity of the Cul1/SkpA/Slmb complex in the eye in the Pk GOF assay. We used RNAi to knockdown components of the complex temporally during establishment of PCP in the eye. Co-expressing the respective RNAi constructs, enhanced the Pk gain-of-function effects (Fig 6A–6E and Suppl. S7D Fig; in control animals, the SCF LOF alone did not cause chirality defects; Suppl. S7A–S7C Fig). As with *nmo* LOF alleles, knockdown of each SCF component caused an increase in chirality defects, and symmetrical clusters in particular, in the Pk GOF scenario. Knockdown of *slmb* also caused a severe loss of photoreceptors, as well as many symmetrical clusters (Suppl. S7D Fig) and therefore the effect was confirmed by analyzing *sev*>*Pk* in a *slmb*^*00295/+*^ background (Fig 6D). For *SkpA*^*IR*^, although there was a reproducible, mild enhancement of the phenotype, there was also loss of photoreceptors and of tissue integrity. Repeating the experiment at higher temperature to increase the knockdown, resulted in lethality (*sev-Gal4* includes the *sev* enhancer coupled to a heat shock promoter), so we were limited in terms of the temperature range in which we could work.

We next examined the effect of reduction in SCF complex function on Pk protein levels. We examined *act*-EGFP-Pk levels in a *slmb*^*00295/+*^ background. Similarly to *nmo*^*DB/+*^ and *GMR*> *DNProsbeta6*, we saw an increase in EGFP-Pk protein levels in the *slmb* LOF background (Fig 6F and Suppl. S7E and S7F Fig).

Taken all together, our data suggest that the increase in chirality defects and symmetrical clusters in the Nmo-Pk phosphorylation context is a result of an altered Pk/Pk-Sple isoform balance, which is caused by reduced Cul1-SkpA-Slmb-mediated proteasomal targeting of Pk.

### Nmo acts on Pk together with dTAK and Hipk

**Fig 7:**
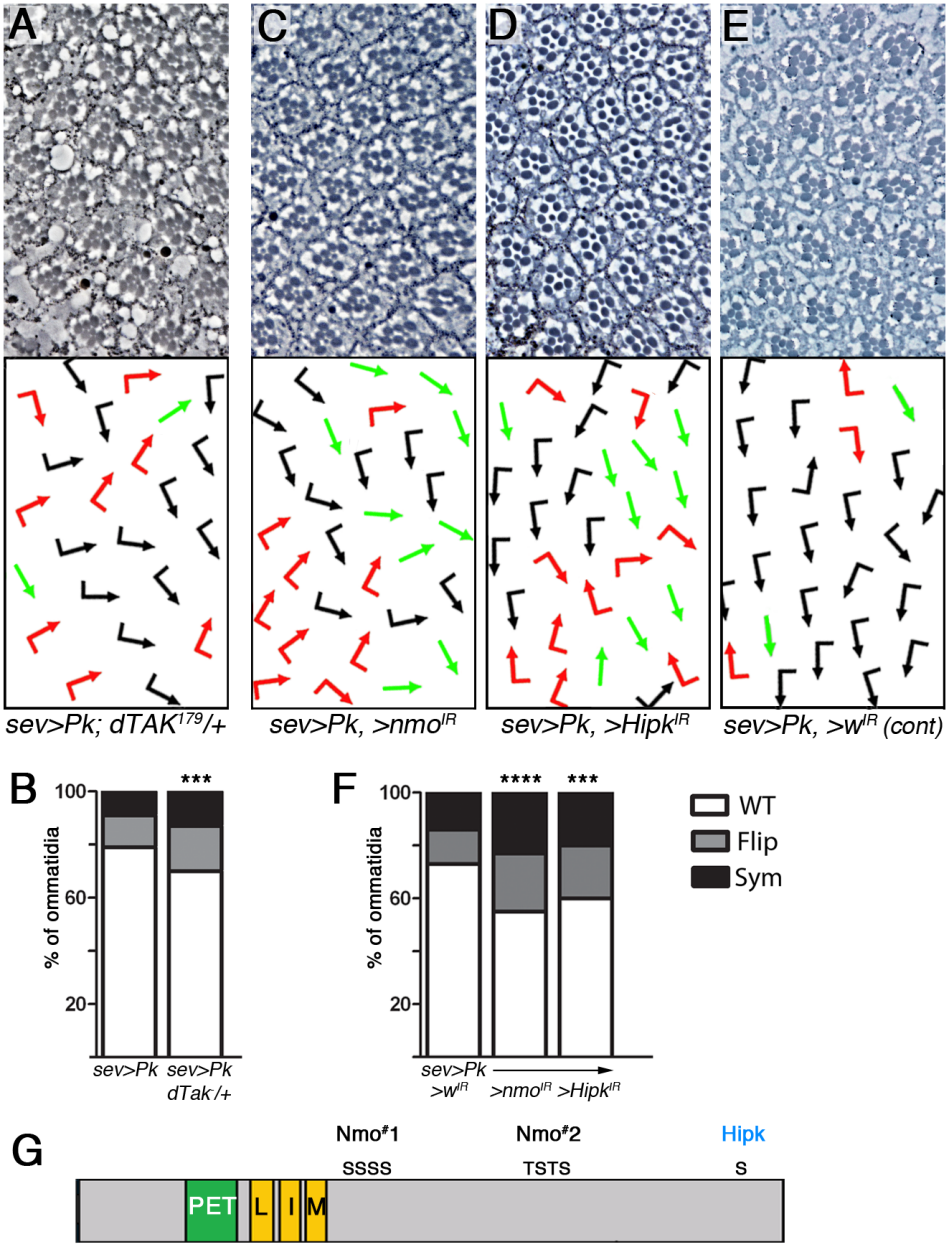
Nmo acts with dTAK and Hipk to regulate Pk. (**A-B**) The phenotype of *sev*>Pk is enhanced in a *dTAK*^*179*^ heterozygous background (**A**), quantified in (**B**) (See also Figs 5 and 6 for *sev*>*Pk* section examples). (**C-E**) Knockdown of *Hipk* (**D**) enhances the *sev*>*Pk*, *>wIR* phenotype (**E**), similarly to knockdown of *nmo* (**C**). (**F**) Quantification of eye phenotypes in **C-E** (in each genotype *n*>200 and *P*<0.005, and <0.0005 for *** and ****, respectively). (**G**) A schematic of the Pk protein showing location of the consensus Hipk phosphorylation site S880, within the consensus HE**S**PSR, along with the two clusters of Nmo phosphorylation sites.

Previous studies have linked Nmo phosphorylation of a substrate to ubiquitination by the SCF complex and the proteasome; in mammalian cells, phosphorylation by Nemo-like kinase (NLK) acting downstream of TGF-β activated kinase (TAK) induces ubiquitination and proteasomal degradation of c-myb [47, 48]. To investigate whether a dTAK-Nmo-protein degradation link is conserved and acts in the Nmo-Pk and PCP-signaling context, we tested for potential effects of the *dTAK*^*179*^ allele [49] on the *sev>Pk* phenotype. The chirality defects induced by *sev>Pk* were indeed enhanced in *dTAK*^*179*/+^ heterozygous females (Fig 7A and 7B), suggesting that dTAK functions upstream of Nmo to limit Pk activity during establishment of PCP. In the TAK1-NLK-SCF complex axis, phosphorylation by Homeodomain Interacting Protein Kinase 2 (HIPK2) also occurs and promotes substrate degradation [47, 48]. We investigated whether the *Drosophila* Hipk homologue was also involved in regulating Pk. Scanning the Pk sequence for potential Hipk consensus sites [50] we detected a putative site in the C-terminus of the protein (Fig 7G). Furthermore, in the Pk GOF assay we noted that knockdown of Hipk enhanced the PCP phenotype, similarly to knockdown of Nmo (Fig 7C–7F). Together these data suggest that Nmo acts with dTAK and Hipk to phosphorylate Pk and recruit the SCF complex, promoting proteasomal degradation of Pk to maintain the Pk/Pk-Sple balance.

### Conclusions

In our systematic, genome-wide screen to identify Nmo substrates during its role in ommatidial rotation, we identified Pk as an unexpected *bona fide* target. Our functional studies then established a role for Nmo kinase during PCP establishment in addition to its known role during the subsequent rotation process. The phosphorylation of the Pk isoform serves as a way to limit the activity of the minor isoform in tissues where Pk-Sple is the major functional isoform. It was previously documented that the isoform balance is regulated at the transcriptional level within the wing, but it was unclear how Pk-Sple was able to act as the ‘major’ isoform in eyes, given that Pk mRNA was even expressed at higher levels [17]. Here we define a novel cell-specific requirement for post-translational regulation of Pk through phosphorylation and associated proteasomal targeting within the R4 cell.

Our results point to a new paradigm of PCP modulation in which spatially-dependent regulation of core PCP protein degradation is required for robust PCP-cell fate coupling. Although it has been well documented that the balance of Pk isoforms is important [15, 17], it has remained unclear as to how such a balance would be maintained and/or reinforced post-translationally. Importantly, the Pk and Pk-Sple isoforms can form different protein complexes and localize to different sites within wing cells [37]. In particular, the correct presence of either Pk or Pk-Sple appears critical in the context of coupling the orientations of the core PCP complex alignments with the Fat/Ds-system polarity orientation [17, 37, 51]. While in the eye Fz-core PCP and Fat/Ds orientation is anti-parallel, the two systems are aligned in a parallel manner in the wing for example [51, 52]. These opposing alignments correlate with differential requirements of either the Pk (wing) or Pk-Sple (eye) isoforms, and hence it is critical to maintain the correct levels of the individual isoforms. Our data suggest that Nmo-mediated phosphorylation of the Pk isoform participates in this context.

Nmo phosphorylation of Pk is required in a tissue and cell-specific manner to maintain low levels of the Pk isoform and favor Pk-Sple in contexts where this is the ‘major’ isoform. Our mosaic analysis demonstrates that Nmo is required in the same photoreceptor cell as Pk-Sple and inhibits Pk function. It has been shown previously that the Cul1/SkpA/Slmb complex regulates overall Pk levels throughout the wing [45, 46], but in this case Pk is the major isoform and optimal Pk levels are required to prevent interference with core PCP complex function, particularly the internalization of transmembrane PCP components. The Cul1/SkpA/Slmb complex appears to play a maintenance role in the wing in preventing Pk hyperactivity throughout the tissue. Interestingly in this case, Pk-Sple accumulated in *Cul1* LOF clones, similarly to Pk [45]. In contrast, our results show that the same machinery operates in a spatially restricted and isoform-specific manner in the eye. How might this specificity be achieved? One possibility emerges from comparison with mammalian studies [47, 48]. In this case, Wnt1 ligand acts upstream of TAK1-NLK-HIPK and subsequent SCF/proteasomal degradation of c-myb. In the case of the developing ommatidia, we observed a requirement for *nmo* in the R4 cell. This is the polar cell of the R3/R4 pair and the one that is closer to the Wingless/dWnt4 ligand sources at the dorsal and ventral poles of the imaginal disc [13, 53]. This raises the possibility that Wingless/dWnt4 are acting upstream of dTAK and Nmo in regulating Pk levels. The involvement of Hipk may be complicated by its pleiotropic roles in regulating Wingless and Hedgehog signaling during eye development, in part through phosphorylation of Slmb itself [54].

This molecular circuitry governing Pk isoform degradation adds another layer of regulation to the intricate feedback mechanisms within a cell to increase robustness of position-based cell fate decisions and helps to coordinate tissue-wide patterning events. Our previous work demonstrated that Vang recruits Nmo to PCP complexes [24]. Based on our current study, this raises the possibility that when Pk, the minor isoform, is recruited into PCP complexes instead of Pk-Sple, Nmo serves a ‘gatekeeping’ role and phosphorylates Pk, targeting it for degradation. The importance of appropriate levels of Pk ubiqutination and degradation is underlined by a recent study that identified USP9X, a de-ubiqutinase, as a regulator of PRICKLE-mediated seizures in mammals [55]. In zebrafish, *nemo-like kinase* (*nlk*) genetically interacts with non-canonical *wnt11* during convergent extension [29], suggesting that Nemo/Nlk regulation of Prickle in PCP patterning processes is conserved. Together, these studies and our work highlight the importance of regulating the balance of Prickle family proteins during embryonic development and adult homeostasis to prevent disease.

## Materials and methods

### Fly stocks and genotypes

The genetic tools and alleles used in this study are listed here along with their Flybase ID (www.flybase.org): *w*^*1118*^ (FBal0018186); *pk*^*pk1*^ (FBal0013838); *pk*^*sple1*^ (FBal0016024); *pk*^*pk-sple13*^ (FBal0060943); *nmo*^*P*^ (FBti0003251); *ey*FLP (FBti0015982); *pw*^+^,*FRT80B* (FBst0001940); *dTAK*^*179*^ (FBst0026275); *Ubx*FLP (FBti0150346); *actGal4* (FBst0003954); *white* RNAi (FBst0031088) *Cul1* RNAi (FBst0029520); *SkpA* RNAi (FBst0028974); *slmb* RNAi (FBst0031056); *slmb*^*00295*^ (FBst0011493); *nmo* RNAi (FBst0025793); *Hipk* RNAi (FBst0035363). *act-EGFP-Pk* and *act-FRT-STOP-FRT-EGFP-Sple* flies, *nmo*^*DB*^, *mδ-lacZ*, and *GMR>DNProsbeta6* (also termed *GMR>Dts5*) flies were generous gifts from David Strutt (University of Sheffield, UK), Esther Verheyen (Simon Fraser University, Canada), Sarah Bray (University of Cambridge, UK), and Hermann Steller (The Rockefeller University, USA), respectively. *sevGal4*, *UAS-Pk*; *sevGal4*, *UAS-Sple* [14, 28]; *sev-Sple-WT* [14, 28]; *UAS-Nmo*, [24], *nubGal4* [25]. *UAS-DNProsβ2 and UAS-DNProsβ6 (also called Dts5)* [42, 56] flies were as described (see refs above).

pUAS attB_myc-Pk-Wt and pUAS attB_myc-Pk-mut1&2 were used to generate transgenic flies in the *y*^*1*^ *w*^*1118*^; *PBac{y*^+^*-attP-9A}VK00027* (FBst0009744) background. Using the specific *AttP* integration site, we ensured that each transgene was expressed from the same genomic locus and thus to a comparable level. Several independent transgenic insertions were tested to confirm comparable phenotypes.

Genotypes for all figures are as follows:

Fig 2 and S2–S4 Figs. **A, G**) *w*^*1118*^ [‘w^-^*’* ‘*wt’*] **B, H**) w^-^;; *nmo*^*P*^ **C, I**) *w*^-^; *pk*^*sple1*^ **D, J**) *w*^-^; *pk*^*sple1*^; *nmo*^*P*^ **E**) *mδ-LacZ* **F**) w^-^; *pk*^*sple1*^; *nmo*^*P*^, *mδ-LacZ* **K**) *w*^-^;; *act-EGFP-Pk/+* **L**) *w*^-^;; *act-EGFP-Pk/nmo*^*DB*^ **S2 B**) *mδ-LacZ* **C**) *y*^-^,*w*^-^, *eyFLP; pk*^*pk1*^; *nmo*^*DB*^, *FRT80B/p[w*^+^*]*, *FRT80B* **D**)*pk*^*pk1*^ **E**) *y*^-^,*w*^-^, *eyFLP; pk*^*pk-sple13*^; *nmo*^*DB*^, *FRT80B/p[w*^+^*]*, *FRT80B* **F**) *w; pk*^*pk-sple13*^ **G**) *w*^-^;; *act-EGFP-Pk/+* **H**) *w*^-^;; *act-EGFP-Pk/nmo*^*DB*^ **S3 A-B**) *w*^-^ **C**) *w*^-^; *pk*^*sple1*^ **D**) *w*^-^;; *nmo*^*P*^ **E**) *w*^-^; *pk*^*sple1*^; *nmo*^*P*^ **F**) *w*^-^;; *act-EGFP-Pk/+* **G**) *w*^-^;; *act-EGFP-Pk/nmo*^*DB*^ **S4 A,C-D**) w; *UbxFLP*/+; *act-FRT-STOP-FRT-EGFP-Sple/+* **B, E**) w; *UbxFLP*/+; *act*-*FRT-STOP-FRT-EGFP-Sple/nmo*^*DB*^ **F**) *w;; sevGal4*, *UAS-Sple/+* **G**) *w;; sevGal4*, *UAS-Sple/UAS-nmo*^*IR*^ **H**) *w;; sevGal4*, *UAS-Sple/nmo*^*DB*^ Fig 3 and S5 Fig. **B**) *w*^-^*/Y;; sevGal4/UAS-mycPk-WT* **C**) *w*^-^*/Y;; sevGal4/UAS-mycPk-mut1&2* **E**) *w*^-^*/Y; sevGal4*, *UAS-Pk/+* **F**) *w*^-^*/Y; sevGal4*, *UAS-Pk/+; nmo*^*P*^*/+* **G**) *w*^-^*/Y; sevGal4*, *UAS-Pk/+; nmo*^*DB*^*/+* **H**) *w*^-^*/Y; sevGal4*, *UAS-Pk/+; UAS-NmoGFP/+* **S5 C**) *w*^-^*/Y; pk*^*sple1*^; *sevGal4/ UAS-mycPk-WT* **D**) top) *w*^-^*/Y; nubGal4/+* middle) *w*^-^*/Y;; nubGal4/UAS-mycPk-WT* bottom) *w*^-^*/Y;; nubGal4/UAS-mycPk-mut1&2* **E**) *sev-Sple-WT/+* **F**) *sev-Sple-MUT1&2/+* **G**) *w; pk*^*pk-sple13*^; *sev-Sple-Mut1&2/+* Fig 4. **A**) *y*^-^, *w*^-^, *eyFLP; pk*^*sple1*^; *nmo*^*DB*^,*FRT80B/p[w*^+^*]*, *FRT80B* Fig 5 and S6 Fig. **A**) For blot *w*^-^: *w*^-^;; *actGal4/UAS-Pk-WT*: *w*^-^;; *actGal4/UAS-Pk-Mut1&2* **B**) For blot *w*^-^: *w*^-^;; *act-EGFP-Pk/+*: *w*^-^;; *act-EGFP-Pk/nmo*^*DB*^ **C**) *w*^-^; *sevGal4*,*UAS-Pk/+* **D**) *w*^-^; *sevGal4*,*UAS-Pk/+; UAS-DNProsβ2/+* **E**) *w*^-^;; *act-EGFP-Pk/+* **F**) *w*^-^;*GMRGal4*, *UAS-DNProsβ6/+; act-EGFP-Pk/+* **H**) For blot *w*^-^;; *act-EGFP-Pk/+* and *w*^-^;*GMRGal4*, *UAS-DNProsβ6/+; act-EGFP-Pk/+* **S6 A**) *w; Ubx-flp/+; act-FRT-STOP-FRT-EGFP-Sple/+* **B**) *w; Ubx-flp/+; act-FRT-STOP-FRT-EGFP-Sple/nmo*^*DB*^ **F**) *w; sevGal4/+; UAS-DNProsβ2/+* **G**) *w*^-^; *GMRGal4*, *UAS-DNProsβ6/+*
Fig 6 and S7 Fig. **A**) *w*^-^; *sevGal4*, *UAS-Pk/+* **B**) *w*^-^*/Y; sevGal4*, *UAS-Pk/+; UAS-Cul1*^*IR*^*/+* **C**) *w*^-^*/Y; sevGal4*, *UAS-Pk/+; UAS-SkpA*^*IR*^*/+* **D**) *w*^-^*/Y; sevGal4*, *UAS-Pk/+; slmb*^*00295*^*/+* **F**) For blot *w*^-^;; *act-EGFP-Pk/+*: *w*^-^;; *act-EGFP-Pk/ slmb*^*00295*^: *w*^-^;*GMRGal4*, *UAS-DNProsβ2/+; act-EGFP-Pk/+* **S7 A**) *w*^-^*/Y; sevGal4/+; UAS-Cul1*^*IR*^*/+* **B**) *w*^-^*/Y; sevGal4/+; UAS-SkpA*^*IR*^*/+* **C**) *w*^-^*/Y; sevGal4/+; UAS-slmb*^*IR*^*/+* **D**) *w*^-^*/Y; sevGal4*, *UAS-Pk/+; UAS-Slmb*^*IR*^*/+* Fig 7A) *w*^-^*/w*^-^, *dTAK*^*179*^; *sevGal4*, *UAS-Pk/+* **B**) *w*^-^*/Y; sevGal4*, *UAS-Pk/+; nmo*^*IR*^*/+* **C**) *w*^-^*/Y; sevGal4*, *UAS-Pk/+; Hipk*^*IR*^*/+* **D**) *w*^-^*/Y; sevGal4*, *UAS-Pk/+; UAS-w*^*IR*^*/+*

### Adult eye sections

Tangential eye sections were prepared as described [57]. Three to ten independent eyes were analyzed per genotype, with over 300 ommatidia scored in each case, except for the mosaic analysis (see below), or as noted in figure legends. Only ommatidia with a full complement of photoreceptors were scored for chirality defects. Rotation defects were not analyzed.χ^2^ or Fisher’s exact test were performed on adult eye data based on the number in each category, depending on which criteria were met regarding sample size and values of each category. Data in graphs are shown as a percentage for clarity.

### Mosaic eye analysis

Animals of the genotype *eyFLP; pk*^*sple1*^; *nmo*^*DB*^*FRT80B/pw+FRT80B* were analyzed. The *white* pigment marks all heterozygous cells and those homozygous for *pw*^+^*FRT80B* following *eyFLP*-mediated recombination. Cells lacking *white* pigment were therefore homozygous for the *nmo*^*DB*^ allele. Tangential eye sections were carefully studied to identify R3/R4 pairs in which one cell lacked pigment (*nmo*^-^ mutant) and the other produced pigment and therefore had at least one functioning copy of *nmo* (*nmo*^*+/+*^ or *nmo*^*+/-*^). Pairs were scored for the number of times the R3/R4 fate decision was correctly resolved (WT) or where PCP defects occurred (flipped or symmetrical clusters), when either R3 had Nmo function or when Nmo function was only present in R4. Over 140 R3/R4 pairs were analyzed from 12 individual adults. Mosaic eye data were analyzed by Fisher’s exact test. For comparison to the *pk*^*sple1*^ and *pk*^*sple1*^; *nmo*^*P*^ mutants, the data from Fig 1 were shown with flips and symmetrical clusters combined into one PCP defect category.

### Leg and wing preparations

Adult legs were dissected and incubated overnight in 70% ethanol. Legs were then washed three times in PBS with 0.1% Triton-X100 and once in PBS. Legs were mounted in 80% glycerol in PBS. The Mann-Whitney U test was used to compare number of tarsal joints. Over 54 legs were analyzed per genotype. Wings were dissected and incubated in PBS with 0.1% Triton-X100 for at least an hour before being mounted in 80% glycerol in PBS.

### Immunofluorescence

Immunofluorescence was performed on third instar larval eye discs and imaged as described [58], using the rabbit anti-beta Galactosidase antibody (Immunology Consultants Laboratory, Inc. 1:200) and mouse anti-Elav antibody (Developmental Studies Hybridoma Bank (DSHB) 1:50).

### Cell culture and western blots

S2 cells were transfected using Qiagen effectene reagent according to manufacturer’s instructions. Cells expressing Myc-Nmo and HA-Pk were lysed and treated in phosphatase assay as described [58]. Eye discs from third instar larvae were dissected and collected in PBS on ice (10 μl per pair of eye discs). An appropriate volume of 5X laemmli sample buffer was added and samples were boiled for 10 minutes. 5–10 disc pairs were loaded per lane of a standard SDS-PAGE gel. Membranes were incubated with anti-myc (Mouse, 1:1000 Santa Cruz Biotechnology), anti-γ-tubulin (Mouse, 1:1000, Sigma), anti-GFP antibody (Mouse, 1:1000 Roche), and anti-Arm (Mouse, 1:20 DSHB) antibody (DSHB). Secondary antibodies and signal visualization were performed using a ChemiDoc MP imager (BioRad, Hercules, CA) as described [58]. The intensity of the EGFP-Pk band was quantified as a signal ratio of myc:γ-tubulin, GFP:γ-tubulin, or GFP:Armadillo antibodies (Arm, *Drosophila* β-catenin, whose levels are unaffected by Nmo [24]). Intensities were measured in ImageJ. Background intensity was subtracted and then the ratio of signal from GFP:Arm calculated. A paired t-test was used to compare the ratio intensities from four independent experiments.

### Cloning

All PCR products were verified by sequencing or by replacing internal fragments with versions from cDNA clones. Gst-Stbm/Vang-Cterm, Gst-Pk-Cterm, Gst-PkΔSacI (N-Δ1 in Fig 1B), Gst-PkΔNruI (N-Δ2 in Fig 1B), pAct_mod_Cterm were described in [28]. pGexKG-EcoCterm (C1 in Fig 1B) was made by cloning the EcoRI fragment of pAct_mod_Cterm [28] into the EcoRI site of pGExKG. pGexKG_Suf (C2 in Fig 1B) was made by re-circularizing SacI digested pGexKG-EcoCterm, thus deleting the C-terminal SacI fragment. pGex4T1_common was cloned by transferring the BamHI fragment of pTopo4.0_Common [28] into the BamHI site of pGex4T1. Topo4.0_Sple_C encoding the Pk^Sple^ specific N-terminal extension contains a PCR product amplified with Sple_C_upper/lower (Sple_C_upper TATGGATCCATGAGCAGCCTGTCAACC, Sple_C_lower ATAGAATTCTCACTCATTTGACTCCTGCTGG). Its insert was then cloned as BamHI/ EcoRI fragment into the corresponding sites of pGex4T1 to give pGex4T1_Sple_C. pBSIISKP motifs (Dom in Fig 1B) contains a PCR product amplified with motifs_upper/lower (motifs_upper TATGGATCCGGCGGACCGCACATGG motifs_lower ATACTCGAGTCACCCCTTGCTGCAGGCG) and encodes the PET and LIM domains of Pk. Its insert was transferred as BamHI/ XhoI fragment into pGex4T1 to give pGex4T1_motifs. pCR-Topo2.1_PET corresponds to the PET domain of Pk that was amplified by PCR using motifs_upper and Pet_lower_XhoI (motifs_upper TATGGATCCGGCGGACCGCACATGG, Pet_lower_XhoI ATACTCGAGTCATCGGGCGCTCATCAGCTG).

Its insert was transferred as BamHI/ XhoI fragment into pGex4T1 to give pGex4T1_PET. pQE31_PkBE (M in Fig 1B) was cloned by inserting a BssHII (blunt)/ EcoRI (blunt) fragment of Pk into the SmaI site of pQE31. pGex4T1_PkBE was cloned as BamHI/ EcoRI fragment that was amplified with PkBssRI_WT_for_Bam and T3XL (PkBssRI_WT_for_Bam TATGGATCCCTGCCGGCGCGCATTCCC, T3XL CGAAATTAACCCTCACTAAAGGGA) into the corresponding sites of pGex4T1.

The eight candidate Nmo sites (Ser/Thr followed by Pro) were mutated to Ala as follows: first, using pBS2SKP_Sple_cloneable [28] as template, ‘mega primers’ were amplified with primers Pk_B/Emut_for_3_BamHI and PK_BE_Mut rev 1, and Pk_BE_Mut for2 and PK_BE_Mut rev 2, respectively. These were used as primers for amplification with PK_BE_Mut rev 2, and Pk_B/Emut_for_3_BamHI, respectively (Pk_B/Emut_for_3_BamHI TATGGATCCCTGCCGGCGCGCATTCCCAGCAGCCACGCCTCCAGCGCACCGCCCATGGCACCGCAACAGCAGCAGCAG, PK_BE_Mut rev 1 CTGGAAGTCGCCGGGTGCGTTCAGAGGCGCTAGGTTCTGCGAGGT, Pk_BE_Mut for2 GCCCGCTCCCAACTTGAGCGTGGCTTCCACCGCCTTGCCGCCAGAGCTTATGGGCGCCCCCACCCACTCGGCGGGCGACAGGTCGCTGAACGCGCCCATG, PK_BE_Mut rev 2 GTCCGGAATTCCCTCGAAGCGCACGCCCTTCTTCTTGGCCGGCTCCCCGCTCATCGGCGCGGAGGAGGA).

The final fragment used further corresponds to a BssHII/ EcoRI fragment of Pk with an added upstream BamHI site in pBS2SKP (after correcting PCR mistakes). Note that the 8 mutations roughly cluster in two regions that are partitioned in two groups of 4 by a SphI site (the first and second cluster are marked by a NcoI or a NarI site, respectively).

The BamHI/ EcoRI insert of pBS2SKP_PkBE_mut was inserted into the corresponding site of pGex4T1 to give Gex4T1_PkBE_mut. Plasmids encoding Gst-Pk versions with one mutant cluster only each were generated by replacement of the other cluster with wild-type SphI/ EcoRI and BamHI/ SphI fragments from pGex4T1_PkBE, respectively, to give Gex4T1_PkBE_mut-first and Gex4T1_PkBE_mut-second.

pFastBacHisC_Nmo was cloned by inserting a BamHI/ PstI fragment of pEGFPN3-Nmo into the appropriate sites of pFastBacHisC. Baulovirus recombination and protein expression and purification were done as described [25]. Construction, expression, and purification of Rok^Cat^ was described in [25] and recombinant Mst1/Mst2 were from Invitrogen [26].

Gst and His tag fusion proteins were prepared as in [14, 25, 28]. ^[32]^P kinase assays were done using 1μg of the indicated Gst protein in 20μl reactions using 0.5μl Nmo (about 350 ng) as described [25]. Gel shift kinase assays were done as in [25] using 2μl of ^[35]^S labeled *in vitro* translated proteins (translated from pOT-108-pan (a kind gift of K. Basler, University of Zuerich), pβTH_common [28] and RE63915 (mbs).

pUAS attB_myc-Pk-Wt and pUAS attB_myc-Pk-mut were cloned as follows:

Pk EcoRINtermMycATGF and PKRA 1232R XhoI primers were used to amplify the 5′ region and to add a myc tag (Pk EcoRINterm Myc ATG F AACGCACCATGGAACAAAAACTTATTTCTGAAGAAGATCTGATGGATACCCCAAATCAAATGCC,PKRA 1232 R XhoI CCGCTCGAGAAAGCCGGCGATAGCTGGTG). The 3′ region was digested using SalI and NotI from either pBS2SKP_PkBE (WT) or pBS2SKP_PkBE_mut to generate myc-Pk-WT or myc-Pk-mut, respectively. These fragments were ligated into pUAS attB.

The generation of *sev-Pk*^*Sple*^ flies was as described in [28]. The construct corresponds to the Sple cDNAs [15] cloned as blunted DraI/AseI fragment into the blunted EcoRI site of pKB267PL (modified after [59]). For sev-Sple^Mut^, the Sple-BssHII-EcoRI fragment of pBS2SKP_PkBE_mut (see above) was cloned into the corresponding BssHII (partial digest)/ EcoRI sites of pBS2SKP_Sple_cloneable [28]. From that construct, a AgeI/EcoRI fragment was cloned into the corresponding sites of sev-Pk^Sple^.

## Supporting information

S1 Fig (related to Fig 1). Nmo phosphorylates Pk. (**A**) Purification of baculovirus-expressed Nmo kinase. Nmo kinase is marked by *. (**B-C**) Band-shift assays of S2 cell culture lysates. Cells were transfected with HA-Pk and myc-Nmo expression constructs. WT, but not a kinase dead (KD) Nmo isoform mutant, induced a band shift of HA-Pk (**B**); the band shift is sensitive to phosphatase-treatment (PPase) (**C**).
(JPG)

S2 Fig (related to Fig 2). *nmo* interacts with the Pk isoform in the eye. (**A**) Schematic of photoreceptor arrangement and corresponding arrows used in all adult eye sections. The arrangement of R3/R4 (in grey) gives a chirality to each ommatidium, denoted by black arrow (dorsal) and red arrow (ventral). The line of mirror symmetry between the dorsal and ventral halves of the eye is called the equator. When R3 and R4 are not specified correctly, symmetrical clusters can form, denoted by straight green arrow. (**B**) Image of whole *wt* eye disc shown in Fig 2E. Box denotes position of panel shown in Fig 2E and arrowhead is positioned at the equator. β-gal (*mδ-lacZ* marking R4) is shown in green and Elav (marking all neurons) is in blue. (**C**) *nmo*^*DB*^ clone in a *pk*^*pk1*^ background marked by lack of pigment. Note that despite the rotation defects within the *nmo*^*DB*^ clone (shaded in grey below) there is no effect on chirality. **(D)** A *pk*^*pk1*^ mutant eye, which looks like wild-type (equator marked by organe line in upper panel), is shown for comparison. (**E**) *nmo*^*DB*^ clone in a *pk*^*13*^ (null) background marked by lack of pigment. Note that despite the enhancement of rotation defects in the clone (shaded in grey below) there is no enhancement of *pk*^*13*^ chirality defects. Loss of photoreceptors is marked by an open circle. A *pk*^*13*^ mutant eye is shown for comparison (**F**). See (**I**) for quantification of symmetrical clusters in (**C-F**). (**G-H**) Loss of *nmo* function enhances an overexpression of Pk: *act-EGFP-Pk*, gain-of-function eye phenotype. Chirality defects occur in *act-EGFP-Pk/+* eyes (**G**) and the proportion of defects increases in *act-EGFP-Pk/nmo*^*DB*^ animals (**H**; quantified in panel **M** of Fig 2). (**I**) Quantification of symmetrical clusters within *nmo*^*DB*^ clones and the surrounding control tissue in *pk*^*pk1*^ and *pk*^*13*^ backgrounds. The equivalent experiment in a *pk*^*sple1*^ background is included (see Fig 4 for an example image of *pk*^*sple1*^; *nmo*^*DB*^ clone tissue). There is only an increase in symmetrical clusters in the *pk*^*sple1*^; *nmo*^*DB*^ clone.
(JPG)

S3 Fig (related to Fig 2). *nmo* does not interact with the Pk isoform in the wing. (**A**) Overview of a wild-type adult wing, rectangle outlining the region shown in (**B-G**). There are no wing PCP defects in any of the following genotypes: *wt* (**B**), *pk*^*sple1*^ (**C**), *nmo*^*P*^ (**D**), *pk*^*sple1*^; *nmo*^*P*^ (**E**), *act-EGFP-Pk* (*o/e Pk*)(**F**) or *act-EGFP-Pk (o/e Pk)/nmo*^*DB*^ (**G**).
(JPG)

S4 Fig (related to Fig 2). *nmo* does not interact with the Pk-Sple isoform. (**A-B**) *nmo*^*+/-*^ loss-of-function (LOF) does not affect Pk-Sple overexpression (o/e). *act-EGFP-Sple* eyes look wild-type (**A**), and are not affected by *nmo*^*+/-*^ LOF heterozygosity. (**B**). (**C-E**) *act-EGFP-Sple* wings show wing hair polarity reversals (overview for box position in (**C**), magnified view in (**D**) and this phenotype is not modified by *nmo* LOF (**E**). (**F-I**) *sevenless(sev)Gal4*-driven Sple overexpression phenotype in the eye (**F**) is not affected by reduction of *nmo* function, via RNAi (**G**) or *nmo* mutation (**H**); quantified in panel **I** (*P*>0.05, not significant).
(JPG)

S5 Fig (related to Fig 3). Nmo limits Pk but not Pk-Sple activity. (**A**) Full size radiograph and coomassie-stained gel as shown in Fig 3A (note Nmo autophosphorylation marked by *). (**B-C**) Increasing the amount of Pk in *pk*^*sple1*^ mutants enhances chirality defects, particularly the proportion of symmetrical clusters (**C**), quantified in (**B** **** *P*<0.0001). This effect is similar to *nmo LOF* (data from Fig 2 are shown for comparison). (**D**) *nubbin(nub)-Gal4*-driven Pk and Pk^Mut1&2^ expression in the wing. Compared to control (*nub*-Gal4), overexpression of Pk and Pk^Mut1&2^ display similar PCP phenotypes. (**E-G**) Direct *sevenless (sev)*-driven Pk-Sple overexpression (*sev-Sple*) phenotype in the eye (**E**) is not affected by mutation of all eight Nmo phosphorylation sites (Sple^Mut1&2^, **F**) and does not interfere with the function of Pk-Sple, as Sple^Mut1&2^ fully rescues the *pk*^*13*^ (null) phenotype (**G**).
(JPG)

S6 Fig (related to Fig 5). Nmo phosphorylation promotes proteasomal degradation of Pk but not Pk-Sple. (**A-C**) Loss of *nmo* function increases Pk but not Pk-Sple protein level in eye discs. The relative amount of EGFP-Sple protein in a *wt* or *nmo*^*DB/+*^ background was calculated and normalized to γ-tubulin levels. A representative blot is shown in (**A**), the fold change in a *nmo*^*DB/+*^ background is shown for each independent experiment in (**C**). Quantification of fold change increase from each independent experiment for EGFP-Pk is shown in (**B**). (**D-E**) Mutation of Nmo phosphorylation sites or co-expression of dominant negative proteasome components (DNProsβ6) increases Pk protein level in eye discs. Quantification of the fold change in Pk^Mut1&2^ to Pk^WT^ (**D**) or EGFP-Pk in *w*^*1118*^ or *GMR*>DNProsbeta6 backgrounds (**E**) from each independent experiment. (**F-G**) Expression of dominant negative proteasome components does not cause chirality phenotypes in the eye: *sevenless*>DNProsβ2 (**F**) and *GMR*>DNProsβ6 (**G**.) (**H-J**) Full length blots from Fig 5A(**H**), 5B(**I**) and 5H(**J**). Size markers (in kDa on left). Note non-specific bands (*) in the γ-tubulin blots, γ-tubulin is indicated with a line, in **H** and **J**. Three different exposures are shown in (**I**) and the red color in the Armadillo blot indicates saturation.
(JPG)

S7 Fig (related to Fig 6). The Cul1/SkpA/Slmb SCF complex promotes Pk degradation. (**A-C**) Knockdown of SCF components on its own does not cause chirality defects in the eye: *sev*>Cul1^IR^ (**A**), *sev*>SkpA^IR^ (**B**), *sev*>slmb^IR^ (**C**). (**D**) Knockdown of *slmb* using RNAi (**D**) enhances the *sev>Pk* gain-of-function phenotype compared to *sev>Pk*, and *w*^*IR*^ control samples (see Figs 6A and 7E). In addition, *sev>Pk*, *>slmb*^*IR*^ causes loss of photoreceptors (marked by black circles in B and D). For quantification and related genotypes see Fig 6E in main text. (**E-F**) Full-length blot (**E**) and quantification of the fold change of EGFP-Pk in *w*^*1118*^ or *slmb*^*+/-*^ backgrounds from independent experiments (**F**) of Fig 6F.
(JPG)
