# Supplementary figures and images for: Prickle is phosphorylated by Nemo and targeted for degradation to maintain Prickle/Spiny-legs isoform balance during planar cell polarity establishment
Source: PLoS Genet. 2018 May 14;14(5):e1007391. doi: 10.1371/journal.pgen.1007391 (PMC5967807; doi:10.1371/journal.pgen.1007391)

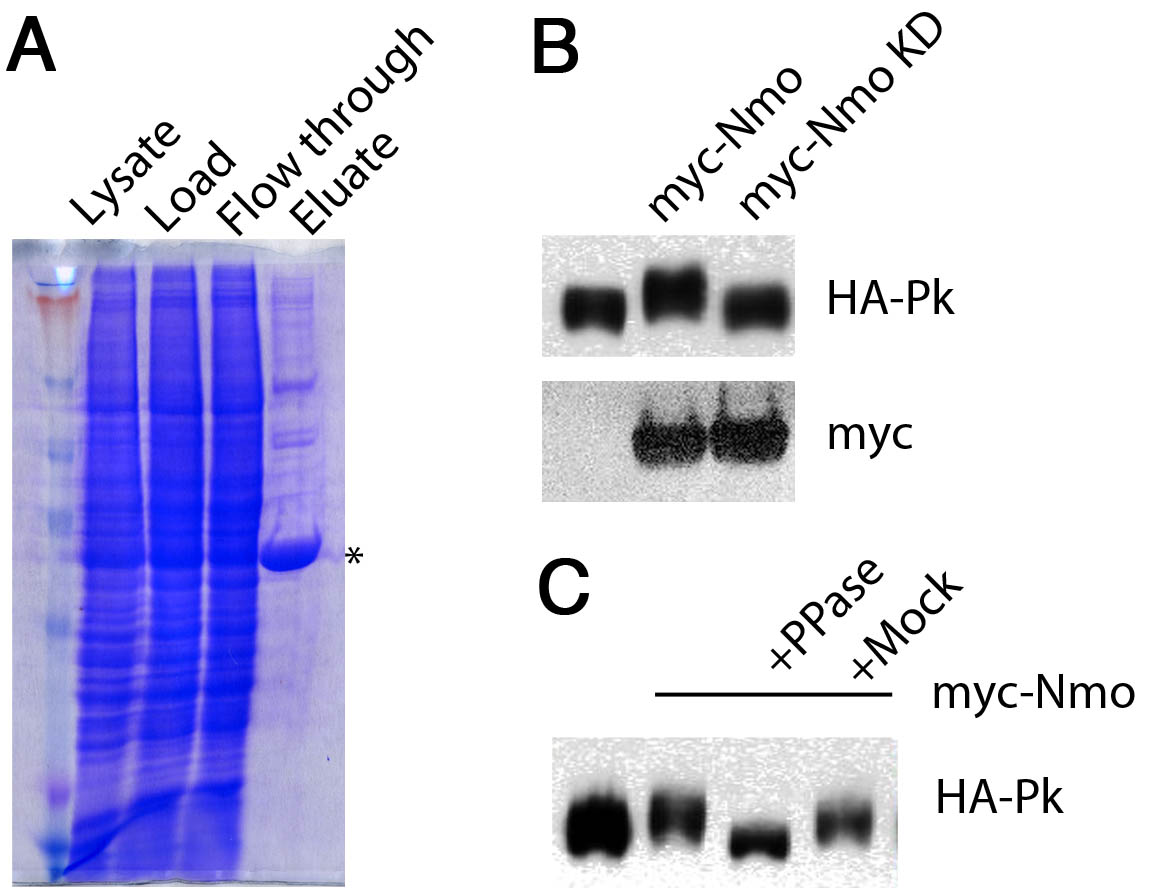

Supplement: S1 Fig — Nmo phosphorylates Pk. (A) Purification of baculovirus-expressed Nmo kinase. Nmo kinase is marked by *. (B-C) Band-shift assays of S2 cell culture lysates. Cells were transfected with HA-Pk and myc-Nmo expression constructs. WT, but not a kinase dead (KD) Nmo isoform mutant, induced a band shift of HA-Pk (B); the band shift is sensitive to phosphatase-treatment (PPase) (C). (JPG) [file pgen.1007391.s001.jpg]

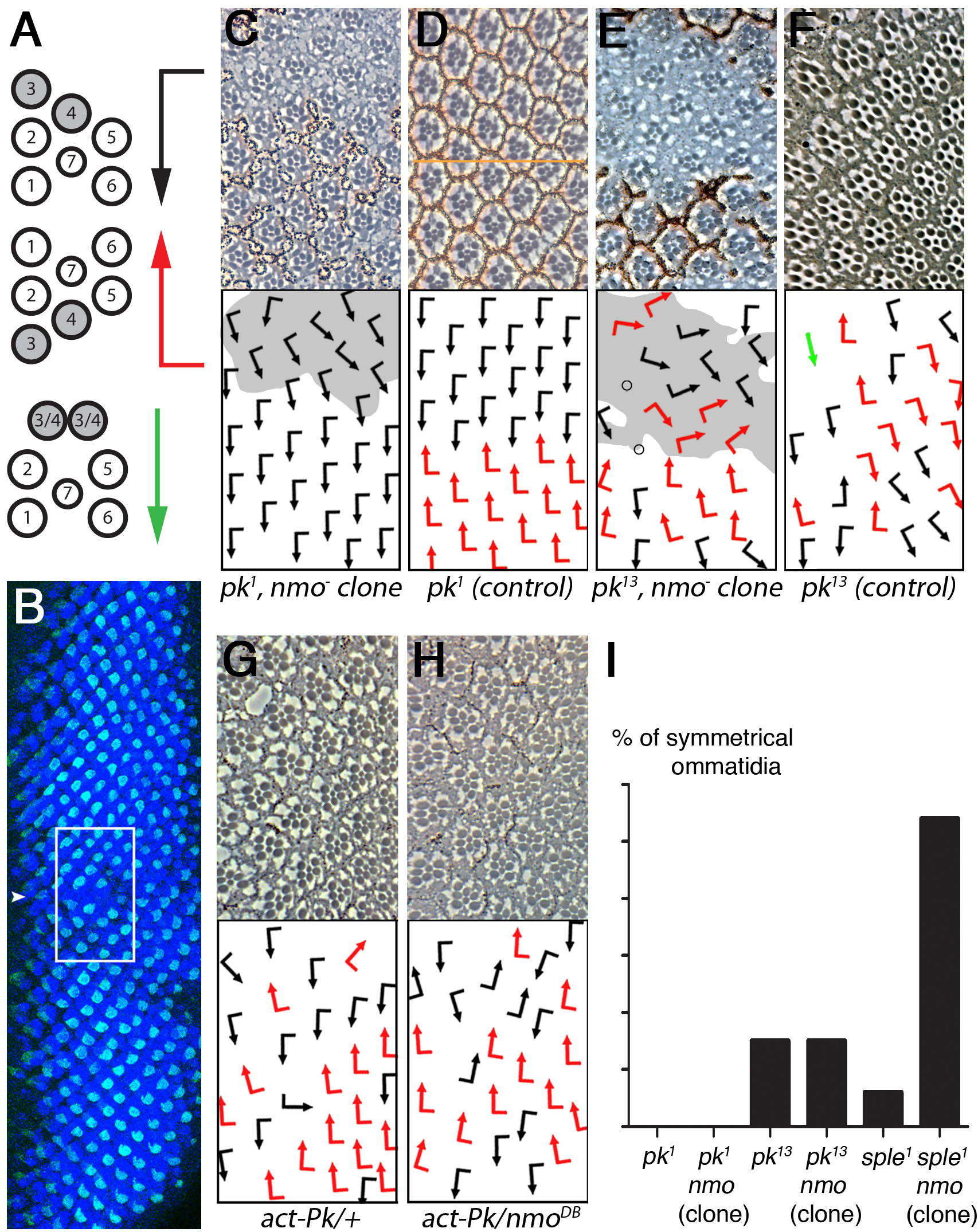

Supplement: S2 Fig — nmo interacts with the Pk isoform in the eye. (A) Schematic of photoreceptor arrangement and corresponding arrows used in all adult eye sections. The arrangement of R3/R4 (in grey) gives a chirality to each ommatidium, denoted by black arrow (dorsal) and red arrow (ventral). The line of mirror symmetry between the dorsal and ventral halves of the eye is called the equator. When R3 and R4 are not specified correctly, symmetrical clusters can form, denoted by straight green arrow. (B) Image of whole wt eye disc shown in Fig 2E. Box denotes position of panel shown in Fig 2E and arrowhead is positioned at the equator. β-gal (mδ-lacZ marking R4) is shown in green and Elav (marking all neurons) is in blue. (C) nmoDB clone in a pkpk1 background marked by lack of pigment. Note that despite the rotation defects within the nmoDB clone (shaded in grey below) there is no effect on chirality. (D) A pkpk1 mutant eye, which looks like wild-type (equator marked by organe line in upper panel), is shown for comparison. (E) nmoDB clone in a pk13 (null) background marked by lack of pigment. Note that despite the enhancement of rotation defects in the clone (shaded in grey below) there is no enhancement of pk13 chirality defects. Loss of photoreceptors is marked by an open circle. A pk13 mutant eye is shown for comparison (F). See (I) for quantification of symmetrical clusters in (C-F). (G-H) Loss of nmo function enhances an overexpression of Pk: act-EGFP-Pk, gain-of-function eye phenotype. Chirality defects occur in act-EGFP-Pk/+ eyes (G) and the proportion of defects increases in act-EGFP-Pk/nmoDB animals (H; quantified in panel M of Fig 2). (I) Quantification of symmetrical clusters within nmoDB clones and the surrounding control tissue in pkpk1 and pk13 backgrounds. The equivalent experiment in a pksple1 background is included (see Fig 4 for an example image of pksple1; nmoDB clone tissue). There is only an increase in symmetrical clusters in the pksple1; nmoDB clone. (JPG) [file pgen.1007391.s002.jpg]

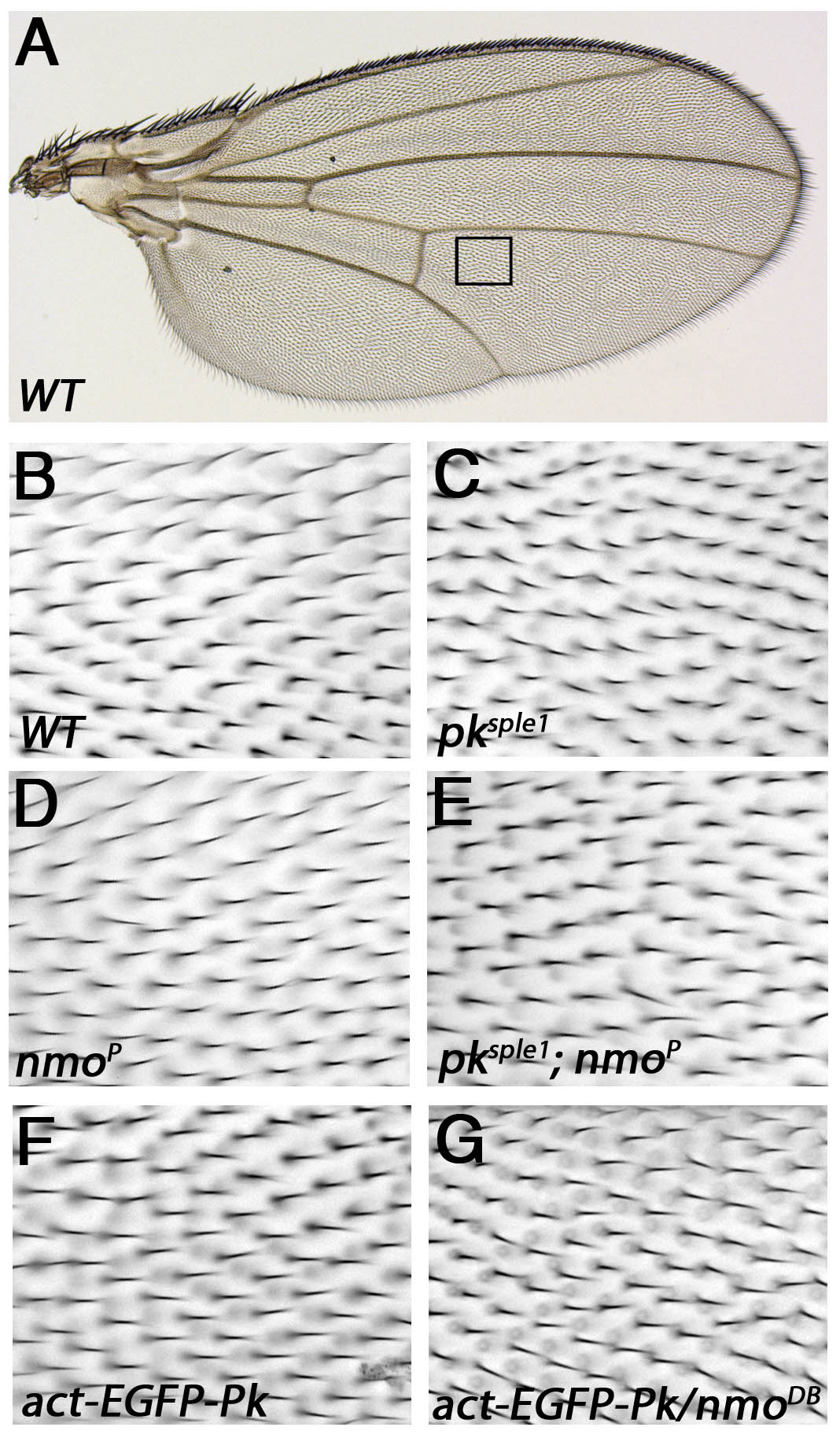

Supplement: S3 Fig — nmo does not interact with the Pk isoform in the wing. (A) Overview of a wild-type adult wing, rectangle outlining the region shown in (B-G). There are no wing PCP defects in any of the following genotypes: wt (B), pksple1 (C), nmoP (D), pksple1; nmoP (E), act-EGFP-Pk (o/e Pk)(F) or act-EGFP-Pk (o/e Pk)/nmoDB (G). (JPG) [file pgen.1007391.s003.jpg]

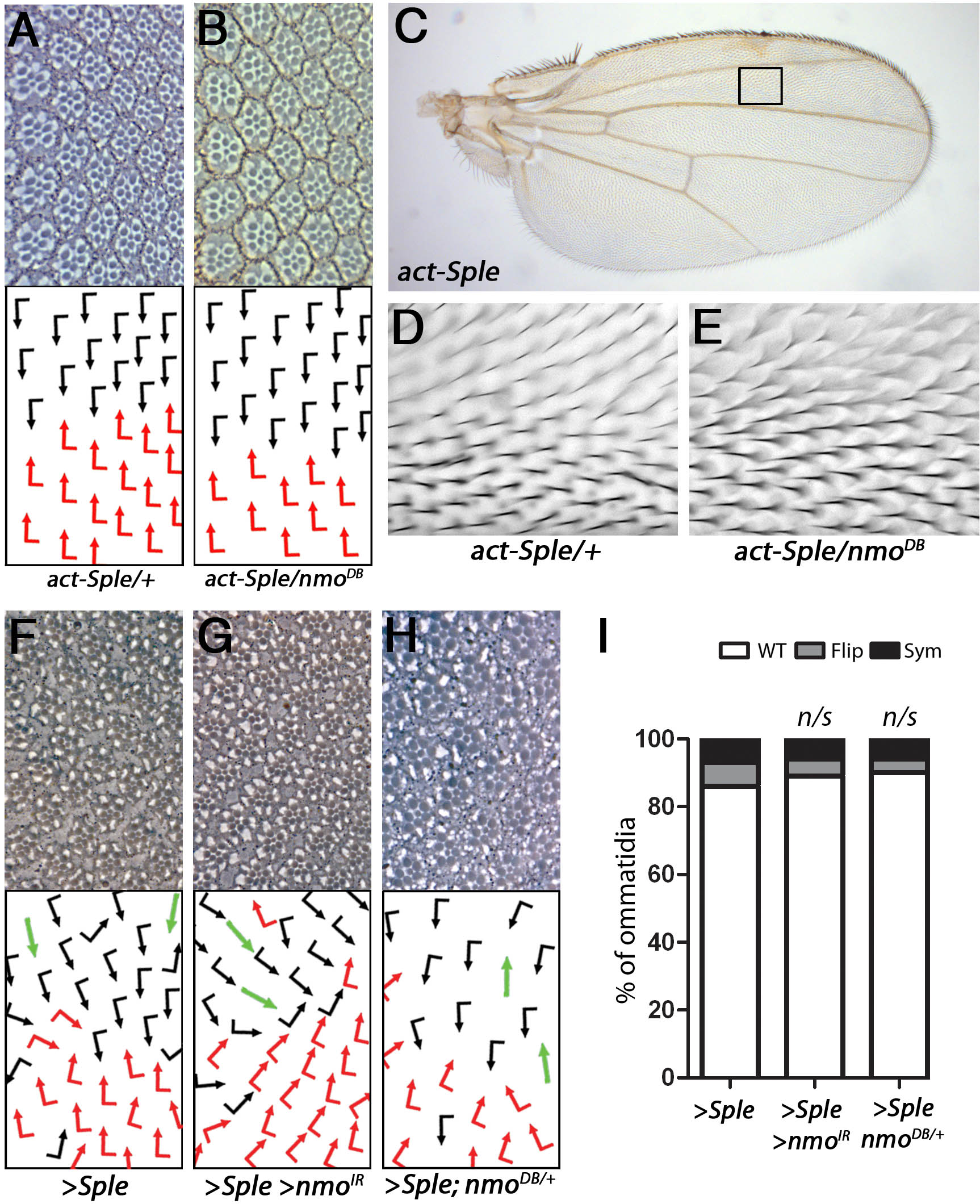

Supplement: S4 Fig — nmo does not interact with the Pk-Sple isoform. (A-B) nmo+/- loss-of-function (LOF) does not affect Pk-Sple overexpression (o/e). act-EGFP-Sple eyes look wild-type (A), and are not affected by nmo+/- LOF heterozygosity. (B). (C-E) act-EGFP-Sple wings show wing hair polarity reversals (overview for box position in (C), magnified view in (D) and this phenotype is not modified by nmo LOF (E). (F-I) sevenless(sev)Gal4-driven Sple overexpression phenotype in the eye (F) is not affected by reduction of nmo function, via RNAi (G) or nmo mutation (H); quantified in panel I (P>0.05, not significant). (JPG) [file pgen.1007391.s004.jpg]

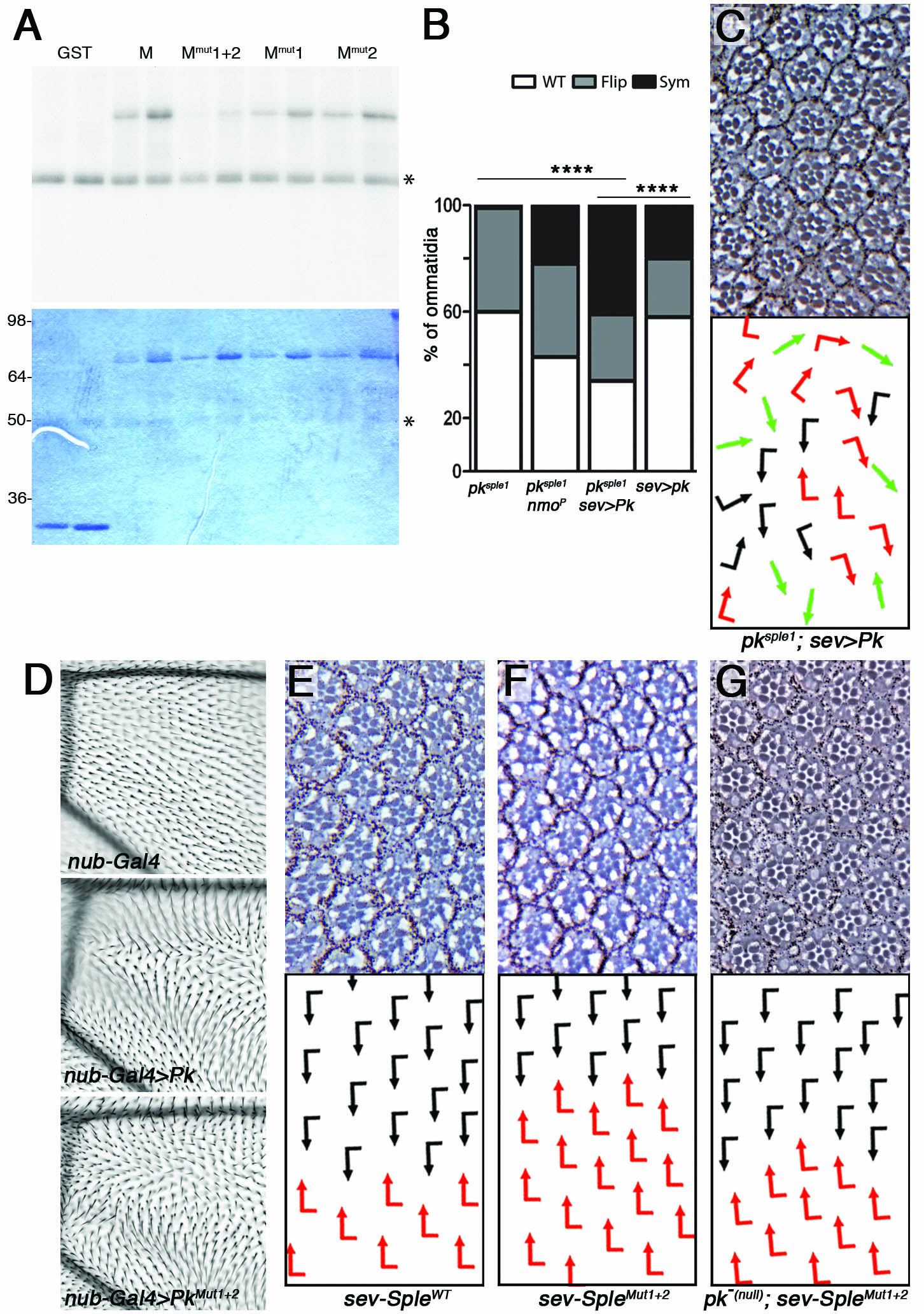

Supplement: S5 Fig — Nmo limits Pk but not Pk-Sple activity. (A) Full size radiograph and coomassie-stained gel as shown in Fig 3A (note Nmo autophosphorylation marked by *). (B-C) Increasing the amount of Pk in pksple1 mutants enhances chirality defects, particularly the proportion of symmetrical clusters (C), quantified in (B **** P<0.0001). This effect is similar to nmo LOF (data from Fig 2 are shown for comparison). (D) nubbin(nub)-Gal4-driven Pk and PkMut1&2 expression in the wing. Compared to control (nub-Gal4), overexpression of Pk and PkMut1&2 display similar PCP phenotypes. (E-G) Direct sevenless (sev)-driven Pk-Sple overexpression (sev-Sple) phenotype in the eye (E) is not affected by mutation of all eight Nmo phosphorylation sites (SpleMut1&2, F) and does not interfere with the function of Pk-Sple, as SpleMut1&2 fully rescues the pk13 (null) phenotype (G). (JPG) [file pgen.1007391.s005.jpg]

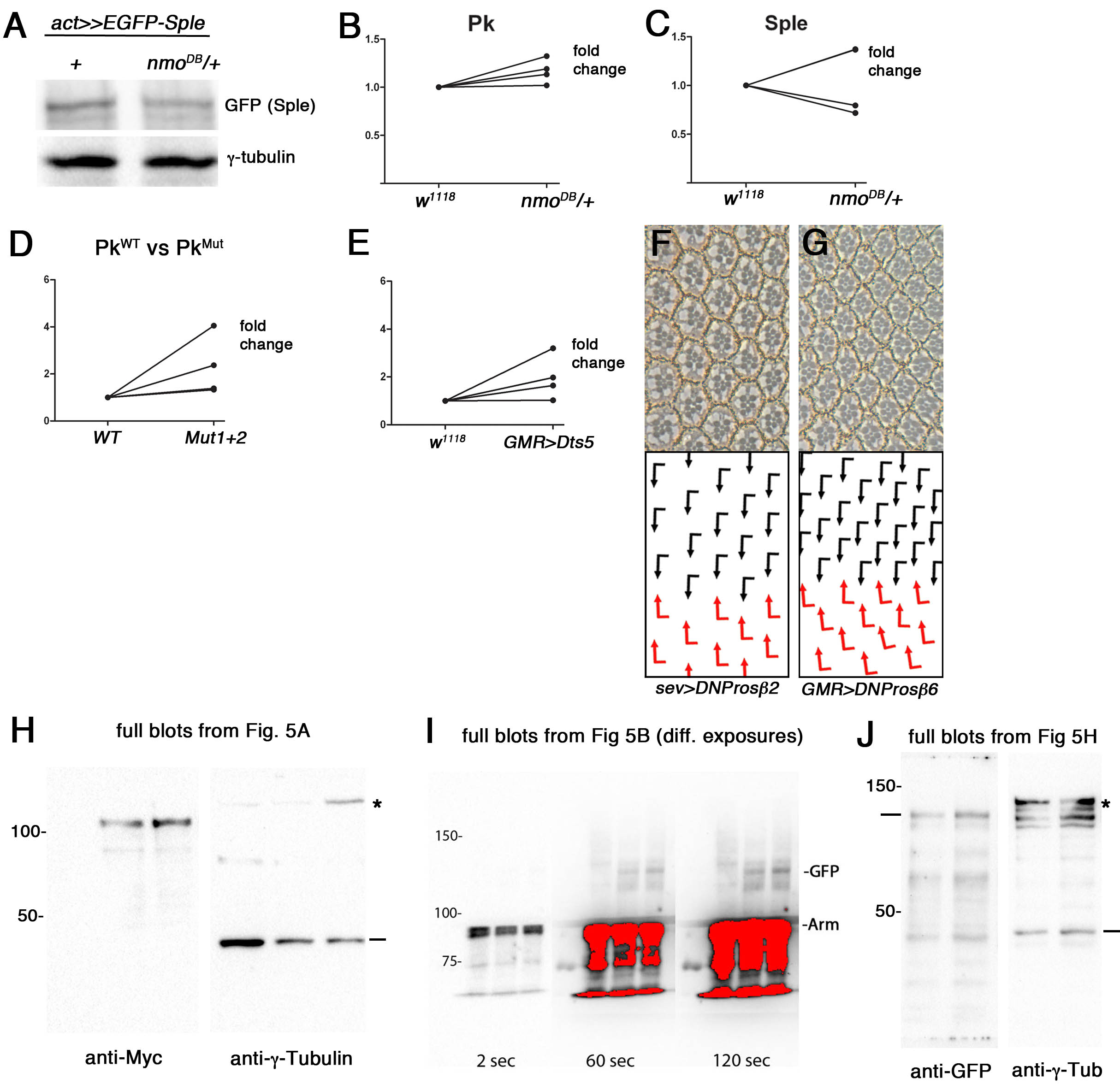

Supplement: S6 Fig — Nmo phosphorylation promotes proteasomal degradation of Pk but not Pk-Sple. (A-C) Loss of nmo function increases Pk but not Pk-Sple protein level in eye discs. The relative amount of EGFP-Sple protein in a wt or nmoDB/+ background was calculated and normalized to γ-tubulin levels. A representative blot is shown in (A), the fold change in a nmoDB/+ background is shown for each independent experiment in (C). Quantification of fold change increase from each independent experiment for EGFP-Pk is shown in (B). (D-E) Mutation of Nmo phosphorylation sites or co-expression of dominant negative proteasome components (DNProsβ6) increases Pk protein level in eye discs. Quantification of the fold change in PkMut1&2 to PkWT (D) or EGFP-Pk in w1118 or GMR>DNProsbeta6 backgrounds (E) from each independent experiment. (F-G) Expression of dominant negative proteasome components does not cause chirality phenotypes in the eye: sevenless>DNProsβ2 (F) and GMR>DNProsβ6 (G.) (H-J) Full length blots from Fig 5A(H), 5B(I) and 5H(J). Size markers (in kDa on left). Note non-specific bands (*) in the γ-tubulin blots, γ-tubulin is indicated with a line, in H and J. Three different exposures are shown in (I) and the red color in the Armadillo blot indicates saturation. (JPG) [file pgen.1007391.s006.jpg]

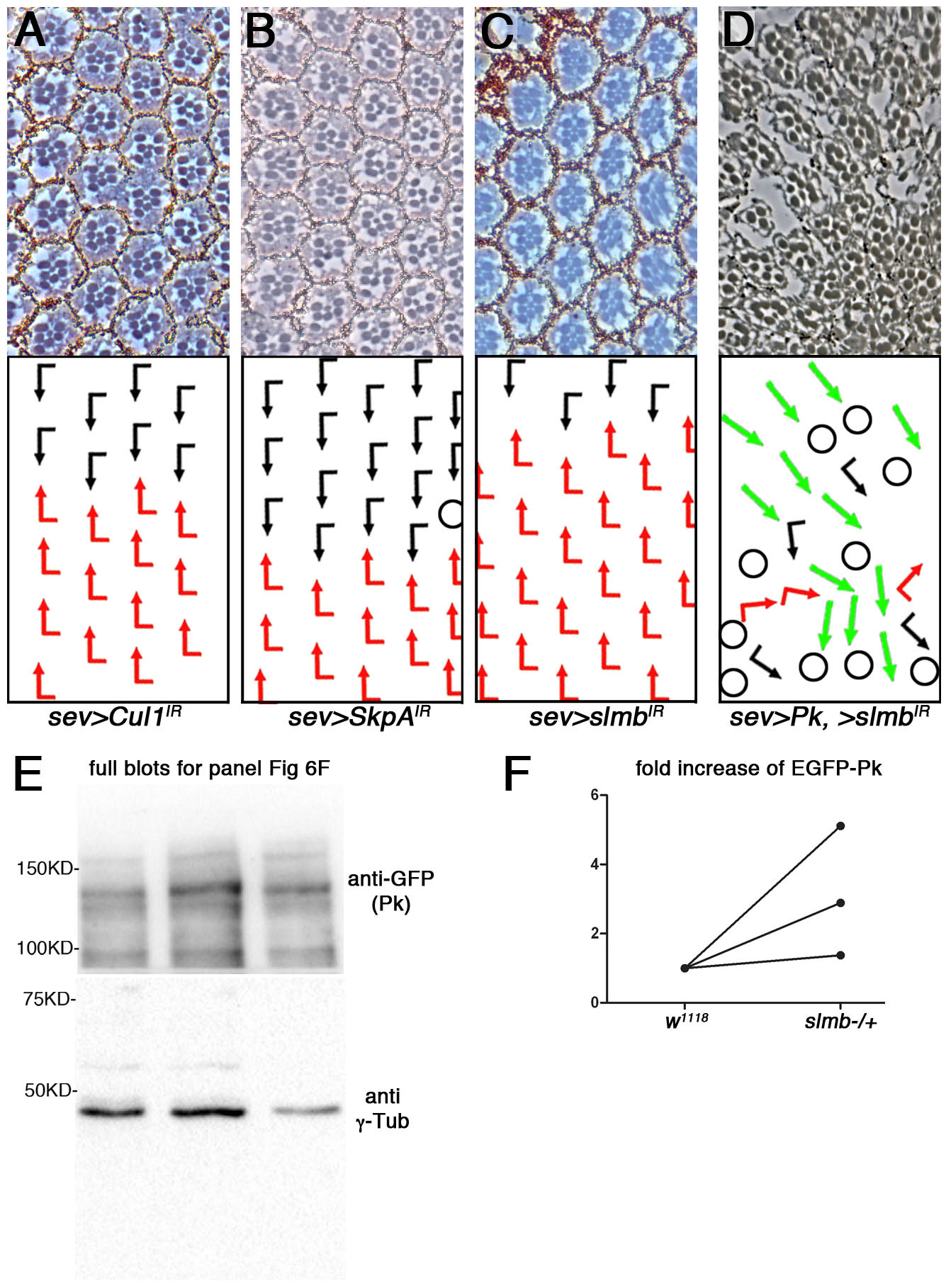

Supplement: S7 Fig — The Cul1/SkpA/Slmb SCF complex promotes Pk degradation. (A-C) Knockdown of SCF components on its own does not cause chirality defects in the eye: sev>Cul1IR (A), sev>SkpAIR (B), sev>slmbIR (C). (D) Knockdown of slmb using RNAi (D) enhances the sev>Pk gain-of-function phenotype compared to sev>Pk, and wIR control samples (see Figs 6A and 7E). In addition, sev>Pk, >slmbIR causes loss of photoreceptors (marked by black circles in B and D). For quantification and related genotypes see Fig 6E in main text. (E-F) Full-length blot (E) and quantification of the fold change of EGFP-Pk in w1118 or slmb+/- backgrounds from independent experiments (F) of Fig 6F. (JPG) [file pgen.1007391.s007.jpg]
